# The origins and genomic diversity of American Civil War Era smallpox vaccine strains

**DOI:** 10.1186/s13059-020-02079-z

**Published:** 2020-07-20

**Authors:** Ana T. Duggan, Jennifer Klunk, Ashleigh F. Porter, Anna N. Dhody, Robert Hicks, Geoffrey L. Smith, Margaret Humphreys, Andrea M. McCollum, Whitni B. Davidson, Kimberly Wilkins, Yu Li, Amanda Burke, Hanna Polasky, Lowell Flanders, Debi Poinar, Amogelang R. Raphenya, Tammy T. Y. Lau, Brian Alcock, Andrew G. McArthur, G. Brian Golding, Edward C. Holmes, Hendrik N. Poinar

**Affiliations:** 1 Department of Anthropology, McMaster University, Hamilton, L8S 4L9 Canada; 2 Department of Biology, McMaster University, Hamilton, L8S 4L9 Canada; 3 Present address: Arbor Biosciences, Ann Arbor, MI 48103 USA; 4 Marie Bashir Institute for Infectious Diseases and Biosecurity, School of Life and Environmental Sciences and School of Medical Sciences, University of Sydney, Sydney, NSW 2006 Australia; 5 Mütter Research Institute, Philadelphia, PA 19103 USA; 6 Mütter Museum of The College of Physicians of Philadelphia, Philadelphia, PA 19103 USA; 7 Department of Pathology, University of Cambridge, Cambridge, CB2 1QP UK; 8 Department of History, Duke University, Durham, NC 27708 USA; 9 U.S. Centers for Disease Control and Prevention, Division of High-Consequence Pathogens and Pathology, Poxvirus and Rabies Branch, Atlanta, GA 30333 USA; 10 Present address: BC Cancer Research Centre, Vancouver, V5Z 1G1 Canada; 11 M.G. DeGroote Institute for Infectious Disease Research, Department of Biochemistry and Biomedical Sciences, DeGroote School of Medicine, McMaster University, Hamilton, L8S 4K1 Canada; 12 Department of Biochemistry, McMaster University, Hamilton, L8S 4L9 Canada

**Keywords:** Vaccination, Smallpox, Vaccinia virus, Ancient DNA

## Abstract

Vaccination has transformed public health, most notably including the eradication of smallpox. Despite its profound historical importance, little is known of the origins and diversity of the viruses used in smallpox vaccination. Prior to the twentieth century, the method, source and origin of smallpox vaccinations remained unstandardised and opaque. We reconstruct and analyse viral vaccine genomes associated with smallpox vaccination from historical artefacts. Significantly, we recover viral molecules through non-destructive sampling of historical materials lacking signs of biological residues. We use the authenticated ancient genomes to reveal the evolutionary relationships of smallpox vaccination viruses within the poxviruses as a whole.

## Background

Smallpox epidemics were caused by variola virus (VARV), a human-specific member of the *Orthopoxvirus* (OPXV) genus of the *Poxviridae*, and resulted in high mortality and morbidity with survivors frequently disabled or disfigured [1–3]. Smallpox remains the only human infectious disease eradicated, a global accomplishment achieved through widespread coordinated vaccination [2, 3]. Despite these profound public health benefits, the origins and diversity of the viruses used in the early vaccination programs remain uncertain. The World Health Organization’s success in eradicating smallpox using vaccinia virus (VACV) (1980) was in part due to the broad protective immunity induced by infection with one OPXV against subsequent infection by another.

The lack of standardisation in vaccination practices and propagation throughout most of its history means that historical vaccine strains may be any one of several OPXVs. On the basis of Edward Jenner’s work [4], cowpox virus (CPXV) was assumed to have been involved in historical vaccination, although horsepox virus (HSPV) and ‘equination’ are also cited [2, 4–7]. Both are thought to produce comparatively self-limiting infections in humans with negligible mortality rates [1, 8]. However, ‘cowpox’ and ‘horsepox’ are likely misnomers, for neither cows nor horses are considered the natural reservoirs of these viruses, and the absence of endemic CPXV or HSPV outside of Europe suggests geographically restricted hosts [9–11]. In 1939, it was recognised that the smallpox vaccine strains being used in the twentieth century were distinct from CPXV [12, 13] and these VACV strains had become the predominant smallpox vaccines [2, 14–18]. However, both the origin of VACV and its natural host or reservoir are also unknown [19].

Vaccination ‘kits’ and their biological contents (scabs, lymph) provide evidence of early vaccination methods and materials and remain in medical collections/archives across the globe. Kits found in collections relating to the American Civil War correspond to a time of known medical crisis and intervention to prevent smallpox outbreaks [20–23].

To better characterise the origins of smallpox vaccination, we investigated the origin, diversity and propagation of early smallpox vaccine strains by extracting and sequencing total DNA and analysing both the viriome and metagenome from these kits. The results reported herein are an attempt to begin to survey viruses that were in use for smallpox vaccination and circulating in Philadelphia in the mid-to-late nineteenth century, during or just after the conclusion of the American Civil War.

## Results and discussion

**Fig. 1:**
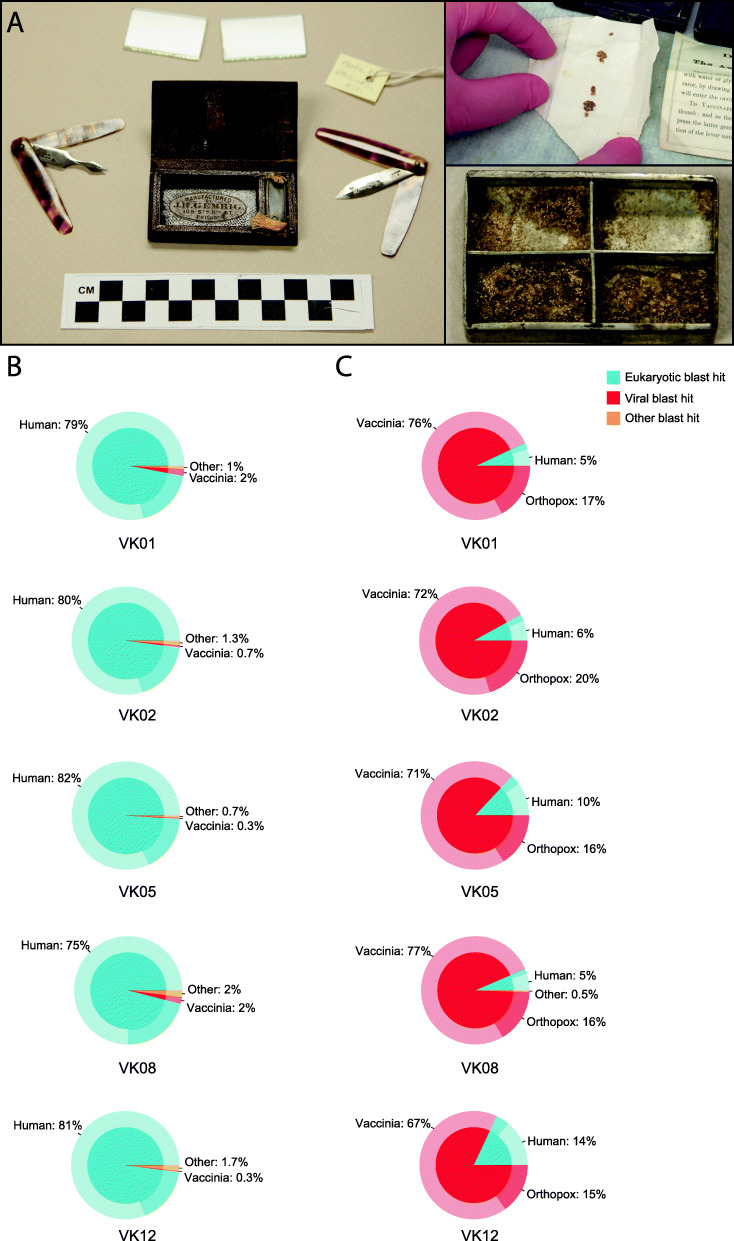
Mütter Museum vaccination kits. **a** Left panel, Mütter catalogue # 17090.29 representative vaccination kit containing two vaccination lancets, a small metal box to hold scabrous material and glass slides to hold lymph. Upper right panel, scabrous material from Mütter catalogue # MISC-1090, subsamples of this material were used to produce library VK01. Lower right panel, metal box from Mutter catalogue # 17831.42.16, it is internally divided into four quadrants. The lower two quadrants are filled with a thick hardened residue, and a portion of the same residue remains in the upper left quadrant. This hardened substance, which we believe may be dried lymph, was used to produce library VK08. Photos courtesy of the Mütter Museum of The College of Physicians of Philadelphia. **b** Relative metagenomic composition of the VK01, VK02, VK05, VK08 and VK12 libraries from shotgun sequencing data and **c** post orthopoxvirus targeted enrichment. Blue portions represent reads classified as eukaryotic in origin, with the proportion specifically identified as human highlighted. Red portions represent reads classified as viral in origin with a distinction between those assigned specifically to VACV and other OPXV. Orange portions represent reads not classified as either eukaryotic or viral. Post blast analysis, assignments were visualised in Krona [24] and simplified as pie charts produced using ggplot2 [25]

We were kindly granted access to five historical kits from the Mütter Museum of the College of Physicians of Philadelphia that date to the mid-to-late nineteenth century (likely circa 1859–1873) and are associated with medical practices of the American Civil War era (Fig. 1a). Of the five vaccination kits, four were leather roll-ups containing one or two folding lancets, small glass plates for mixing lymph (fluid collected from blisters of infected patients [23]), and tin boxes with sliding lids to contain scab (or crust) material (Fig. 1a). The fifth kit only contained ‘The Automatic Vaccinator’, a tool designed for use with lymph or scabs smeared into a mixture on glass plates. Museum records, donor history, and manufacturer data regarding the kits’ contents were used to determine date ranges (Additional File 1: Supplementary Materials and Methods, Fig. 1a, Additional File 2: Table S1). Initial evaluation by the Poxvirus Laboratory at the US Centers for Disease Control and Prevention indicated that there was no presence of VARV but identified OPXV DNA within the materials from three of the kits.

The metagenomic profiles of the shotgun libraries generated from these kits were overwhelmingly eukaryotic, with most reads identified as human (~ 80%), yet contained a significant (0.3–2.0%) proportion of reads mapping to VACV with unexpectedly few bacterial DNA reads (Fig. 1b). Post enrichment for OPXV molecules (Additional File 1: Supplementary Materials and Methods), the roles were reversed, with viral reads representing the majority of the molecules sequenced (Fig. 1c, Additional File 2: Table S1). Importantly, both the endogenous human and viral sequences recovered from these historical artefacts have the characteristic signatures of ancient DNA, that is, short fragment lengths and terminal C to T damage (Additional File 2: Table S1, Additional File 3: Fig. S1).

The total human constituent obtained in the shotgun sequence data enabled us to reconstruct the mitochondrial genomes for the human donors from three of our samples VK01, VK02 and VK08 (Additional File 4: Table S2). The haplogroups H1b, T2b4f and U5b1 are most frequently found in west Eurasia, suggesting that the vaccine donors were likely of European ancestry and not African American even though African American children were frequently used for vaccine propagation in the southern states during the American Civil War [23]. Using an algorithm that compares the ratio of the total number of reads that map to both X- and Y-chromosomes [26], we concluded that VK01 and VK02 were clearly derived from female sources (Additional File 4: Table S2). While the algorithm could not assign a definitive sex for VK08, an order of magnitude more reads mapped to the X-chromosome than to the Y-chromosome suggesting a low-level male contamination in that one sample (Additional File 1: Supplementary Materials and Methods, Additional File 4: Table S2). Thus, these three samples suggest that vaccine propagation was still occurring via human to human transfer.

We de novo assembled a nearly complete virus genome (~ 95%) from our shotgun sequencing reads from the VK01 library that had significant read depth when mapped against the VACV Copenhagen strain reference (Additional File 2: Table S1). This draft genome totalled 184,677 bp in length, approximately 95% the length of most VACV genomes though they vary considerably in length due to terminal repetitive motifs. We believe that our reconstructed contig represents the central core of the VK01 strain and one, perhaps partial, of the inverted terminal repeats. Repetitive regions pose serious difficulties for genome reconstruction from aDNA libraries as read lengths are extremely short and prohibit scaffold-building sections that span these regions [27]. Our other shotgun libraries did not have adequate read length and/or depth for successful de novo assembly (Additional File 2: Table S1, Additional File 5: Table S3). We generated consensus sequences for the remaining four samples by mapping both the shotgun and enriched data to our VK01 contig and calling consensus sequences for positions that had at least 10x coverage with variants present at ≥ 0.9 frequency. This methodology produced consensus sequences for VK02, VK05, VK08 and VK12 that are between 97.0 and 99.9% complete at > 10x coverage relative to the VK01 assembly.

**Fig. 2:**
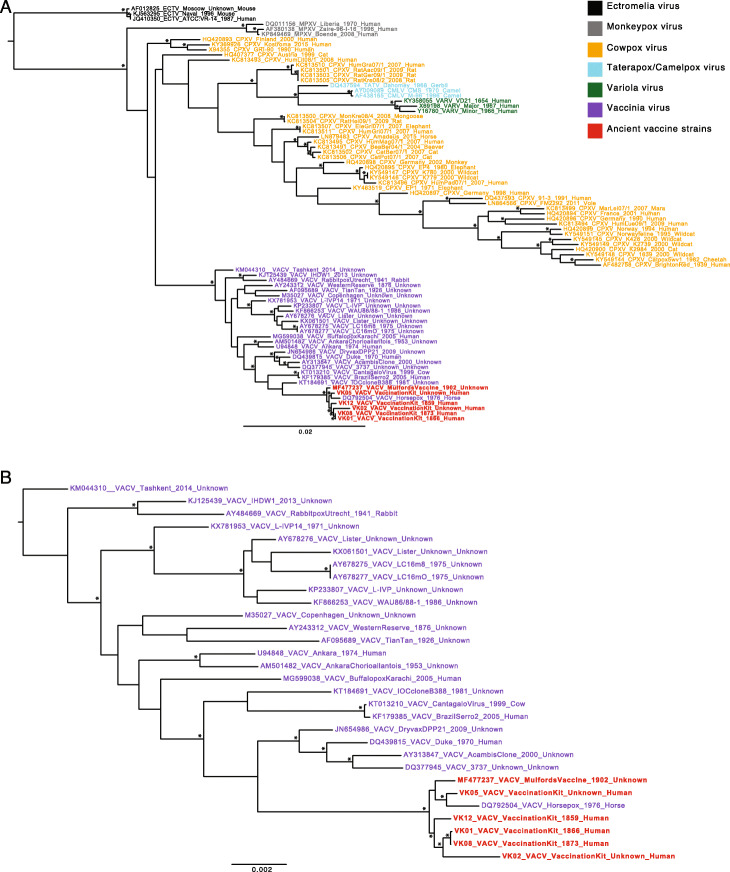
Maximum likelihood phylogenetic analysis of historical vaccine strains in relation to other OPXVs. **a** Position of the VACV clade and Mütter Museum vaccination strains within a broader OPXV phylogeny rooted using Ectromelia (ECTV) as an outgroup. **b** Position of the Mütter Museum vaccination strains within the phylogeny of available VACV strains, rooted using VACV Tashkent KM044310 as an outgroup. Nodes with > 95% bootstrap support are indicated with asterisks. All horizontal branch lengths are scaled according to the number of nucleotide substitutions per site

To determine the evolutionary relationships of the five Mütter vaccine strains, we placed them within an expansive OPXV phylogeny that includes representative viruses described as CPXV, HSPV, VACV and VARV (Fig. 2). The vaccination kit viruses sit firmly within the VACV clade, indicating that VACV was indeed circulating prior to the twentieth century. To ease concerns that the phylogenetic positioning of our consensus sequences was dictated by the reference, VK01, we repeated the mapping process with an additional three VACV reference sequences and generated new consensus sequences; the positioning of VK01, VK02, VK05, VK08 and VK12, within the larger OPXV tree remained unchanged (Additional File 1: Supplementary Materials and Methods, Additional File 6: Fig. S2). Within the VACV clade, all of the vaccination kit strains recovered from the Mütter collection cluster tightly, differing from the VK01 assembly at 20–352 polymorphic positions, suggesting that there may have been little diversity in vaccination strains circulating amongst Philadelphia physicians at this time (Fig. 2). Notably, these strains group closely with a commercially produced vaccination strain from 1902, also manufactured in Philadelphia [6]. Importantly, that the later vaccine strain was sequenced by another group acts to validate the genomic data obtained here. Interestingly, the strain identified as HSPV, isolated from a horse in an 1976 outbreak in Mongolia [28], also clusters closely with these vaccine strains. Given the age of the sample and its phylogenetic position within the larger VACV group, it is more reasonable to re-classify the 1976 Mongolian HSPV isolate as either a VACV vaccine escape strain or a VACV virus introduced into horses from an unknown animal reservoir. Similar occurrences have been noted previously in both buffalo and rabbit [9–11], and this observation fits with the Jenner-era assertion that horsepox was not found outside of Europe [9]. In addition to the paraphyletic nature of strains described as ‘cowpox’, that the ‘horsepox’, ‘buffalopox’ and ‘rabbitpox’ viruses all fall within the VACV clade further demonstrates the imprudence of naming these viruses after the host of isolation (Additional File 7: Fig. S3).

Unfortunately, there is no temporal structure across the OPXV phylogeny, including within the VACV clade, making it impossible to reliably estimate either a rate of nucleotide substitution or divergence times (Additional File 8: Fig. S4). This lack of temporal structure likely reflects the differences in evolutionary rates within different host species [29, 30] and that many of the available genomes have gone through repeated passaging and/or cell culturing after they were originally isolated (Additional File 7: Fig. S3, Additional File 9: Table S4).

Our phylogenetic analysis robustly places the historical vaccination strains within the diversity of viruses labelled ‘vaccinia’. The most closely related strains of VACV include known North American vaccine strains of the twentieth century as well as many strains currently circulating in Brazil, including Cantagalo virus—a virus circulating amongst dairy cows thought to represent a historical escape of smallpox vaccine [31, 32] (Fig. 2, Additional File 7: Fig. S3, Additional File 10: Fig. S5). Although the relationship of the Mütter vaccine strains and the Brazilian and North American vaccine strains varies depending on the inclusion/exclusion of other sequences (Fig. 2), their close relationship to the Cantagalo and IOC strains from Brazil may tentatively suggest a tie to the Beaugency lymph strain of the late nineteenth century that had arrived in the New England area by the 1870s [33].

## Historical context

During the American Civil War (1861–1865), Philadelphia functioned as the second largest hospital city in the northern states, after Washington, D.C. The birthplace of American medicine, Philadelphia fostered the creation in 1787 of the College of Physicians of Philadelphia, a professional-fraternal institution to advance medical practice and research, its members having founded the first hospital and medical school in the USA. The 1860s wartime influx of wounded and sick soldiers constituted such a large and growing population of medical patients that army authorities created specialty hospitals centring on the treatment of particular disorders. The work in these hospitals effectively created medical disciplines of cardiology and neurology. Wartime Philadelphia physicians employed state-of-the-art medical technology, much of it furnished by local firms, as the city had become a leading centre for the manufacture of scientific instruments. The College created the Mütter Museum, which opened during the war in 1863 as a repository of artefacts and specimens for teaching and research. The museum began to acquire vaccination material and related tools at this time. During the war, most of the College members served either in the army or as contract physicians and, as such, administered smallpox vaccination according to standard army protocol. Vaccination was required of all military recruits in northern and southern armies. It is not surprising, therefore, that the Mütter Museum possesses vaccination tools and remnants of early vaccine during Philadelphia’s concentration of medical assets during the war and into the 1870s and 1880s.

## Conclusions

Within the historical context of American medical practices in the 1860s and 1870s, we note that vaccination was a uniquely human process. Vaccination material was still being produced within humans and transferred directly from donors to patients, a process that changed in the following decades in response to public health concerns over iatrogenic disease spread and the for-profit industrialisation of vaccine production through animals. The similarities between the construction of the kits and their contents, which were not available in catalogues but seemingly constructed through a bespoke manner, suggest that there was a common wisdom in how vaccination was practised in this particular era. As part of this project, we surveyed instrument catalogues available in the USA during the last half of the nineteenth century and found no advertised vaccination kits. Instrument firms, however, advertised that custom-built cases would be created to order. Indeed, the similarity in the virus strains, not only from these five Civil War Era kits, but from the 1902 Mulford’s strain [6], suggests that there may have been a common source for material in the Philadelphia area.

This work highlights the value of research involving historical medical collections, by presenting a novel, non-destructive methodology to recover DNA, thereby preserving these artefacts for continued display and study. Indeed, this project was only feasible as a result of the foresight and meticulous and continued conservation of museum collections by dedicated curators and collection management. The clear identification and reconstruction of near-complete genomes of VACV from these vaccination kits, which were in use during the American Civil War era, indicates that these strains were circulating within humans and via physician networks prior to the twentieth century.

## Materials and methods

Five vaccination kits dating to the mid-to-late nineteenth century were found within the collection of the Mütter Museum of the College of Physicians of Philadelphia. The kits were first sent to the US Centers for Disease Control and Prevention in Atlanta, USA, where nine specimens from the five kits were tested with amplification assays to detect VARV [34], contemporary VACV (CDC, unpublished) and generic OPXV sequences [35]. There was no amplification of the VARV-specific or VACV-specific assays. Homogenates of the scabs from Mütter collection # MISC-1090 and # 17090.33 as well as a swab from the glass plates of Mütter collection # 17831.42.16 tested positive for the generic OPXV assay and were further placed into culture: no growth was observed. The kits were then transported to the McMaster Ancient DNA Centre and processed in dedicated clean room facilities through both destructive analysis of organic materials (crusts and lymph) and non-destructive sampling of inorganic materials (lancets, boxes and glass slides). Full details of destructive and non-destructive sampling techniques and sequencing conditions are described in the Supplementary Materials and Methods (Additional File 1).

We attempted to de novo assemble genomes from the pooled shotgun reads of VK01, VK02 and VK08. From the VK01 library, we were able to assemble a 184-kbp contig representing approximately 95% the total length of a VACV (Additional File 5: Table S3). For the remaining libraries, shotgun and enriched datasets were separately mapped with a modified version of BWA (https://github.com/mpieva/network-aware-bwa) [36] to the VK01 de novo contig. Mapped reads from separate sequencing runs and the shotgun and enriched libraries were then filtered of PCR duplicates and restricted to a minimum length of 35 bp and a minimum mapping quality of 30. The endocaller program of schmutzi v1.0 (qual -60) was then used to generate consensus base calls [37]. In addition to reconstructing the genomes of VK01, VK02 and VK08, we also produced the mitochondrial genomes of their hosts [37–39] and further attempted to determine the sex of the human hosts using an algorithm developed specifically for shotgun sequencing data from ancient human remains [26].

The five Mütter viral genomes were aligned with 76 representative OPXV genomes including ectromelia virus as an outgroup (Additional File 9: Table S4) using MAFFT v7.205 [40]. The resultant alignment (263,227 bp) was cleaned of poorly aligned regions and indels using Gblocks v0.91b [41] and subsequently utilised as the input for a maximum likelihood phylogeny (134,607 bp) using PhyML [42] (Fig. 2). Metadata including host of virus isolation was overlain on the ML phylogeny and visualised using Grapetree [43] (Additional File 7: Fig. S3). The ML phylogeny produced from the data set including the additional 76 OPXV genomes, as well as those restricted to the VACV clade, were used as input along with either year of strain collection (if known) or year of genome sequencing in root-to-tip regressions on the ML trees to determine the extent of temporal structure in the data and hence the level of support for a molecular clock of evolutionary change (Additional File 8: Fig. S4). Complete details of computational methods are further described in the Supplementary Materials and Methods (Additional File 1).

## Supplementary information

**Additional file 1.** Supplementary Material and Methods.

**Additional file 2: Table S1.** Details of the libraries generated from the Mütter Museum vaccination kits and vaccinia virus mapping statistics.

**Additional file 3: Figure S1.** Terminal damage rates for VK01, VK02, VK05, VK08 and VK12 libraries mapped to VACV strain Copenhagen reference (M35027) and human mitochondrial reference (rCRS).

**Additional file 4: Table S2.** Mapping statistics for human component of libraries VK01, VK02 and VK08.

**Additional file 5: Table S3.** Summary statistics produced by QUAST for SPAdes de novo assemblies of VK01, VK02 and VK08 from shotgun data.

**Additional file 6: Figure S2.** Maximum likelihood trees for Mutter vaccine strains called in reference to alternative VACV genomes.

**Additional file 7: Figure S3.** GrapeTree analysis of 79 OPXV.

**Additional file 8: Figure S4.** Regression analyses of root-to-tip genetic distance on the ML phylogeny against either year of collection or year of sequencing.

**Additional file 9: Table S4.** OPXV genomes used for comparison in the phylogenetic analyses.

**Additional file 10: Figure S5.** Partitioned maximum likelihood analysis of OPXV.

**Additional file 11: Figure S6.** Mütter catalogue # 17090.29 before and after non-destructive sampling.

**Additional file 12: Table S5.** OPXV genomes used for bait design.

**Additional file 13: Figure S7.** Comparison of terminal damage patterns of VK01.

**Additional file 14: Table S6.** BLASTX, Bowtie2, and taxonomic *k*-mer annotation of VK02 reads against CARD reference sequences.

**Additional file 15: Figures S8–10.** VK01 coverage relative to Copenhagen VACV strain M35027, GRI90 CPXV strain X93455, and Horsepox strain DQ792504.

**Additional file 16: Figure S11.** Maximum likelihood phylogenetic analysis of historical vaccine strains in relation to other OPXV using largest de novo assembled contig for each sample.

**Additional file 17.** Review history.

## Data Availability

The de novo assembly for VK01 is available on GenBank, accession MN369532 (63). Complete sequencing data are available through the SRA accession PRJNA561155 (64).
