# Supplementary material for: The origins and genomic diversity of American Civil War Era smallpox vaccine strains
Source: Genome Biol. 2020 Jul 20;21:175. doi: 10.1186/s13059-020-02079-z (PMC7370420; doi:10.1186/s13059-020-02079-z)
Supplement: Supplementary file 1 — Additional file 1. Supplementary Material and Methods. [file 13059_2020_2079_MOESM1_ESM.docx]

**Title:** The origins and genomic diversity of American Civil War Era smallpox vaccine strains

**Authors:** Ana T. Duggan, Jennifer Klunk, Ashleigh F. Porter, Anna N. Dhody, Robert Hicks, Geoffrey L. Smith, Margaret Humphreys, Andrea M. McCollum, Whitni B. Davidson, Kimberly Wilkins, Yu Li, Amanda Burke, Hanna Polasky, Lowell Flanders, Debi Poinar, Amogelang R. Raphenya, Tammy T.Y. Lau, Brian Alcock, Andrew G. McArthur, G. Brian Golding, Edward C. Holmes and Hendrik N. Poinar

**Supplementary Materials and Methods**

**Mütter Collection details**

Mütter catalogue # MISC-1090, a small portion of a scab, wrapped in paper, placed inside a Codman & Shurtleff “automatic vaccinator” case from 1866 produced library VK01 (Additional File 2: Table S1). The kit belonged to Dr. William Pepper Jr. (1843-1898), a leading University of Pennsylvania educator. We used 1866, the year of case production, as the sampling date for VK01 in phylogenetic analyses.

Mütter catalogue # 17090.35 is a kit of unknown provenance and produced libraries VK02 and VK03 (Additional File 2: Table S1)­. No contextual information is known for this object, VK02 was omitted from regression analyses requiring a sampling date.

Mutter catalogue # 17090.33 produced libraries VK05 and VK06 (Additional File 2: Table S1). No contextual information is known for this object, VK05 was omitted from regression analyses requiring a sampling date.

Mutter catalogue # 17831.42.16 a kit containing two thumb lancets “with tortoiseshell guards, both with ‘Charriere’ stamped on the blade; guards are much worn, not identical; on one are four evenly-spaced horizontal shallow cuts, on other are three similar cuts,” according to museum records produced, libraries VK08 and VK09 (Additional File 2: Table S1). This vaccination kit belonged to Dr. Jacob Mendez DaCosta (1833-1900), a pioneering Philadelphia physician who, during the Civil War, supervised a special hospital ward treating cases of heart disease caused by combat stress. DaCosta practiced in Philadelphia from 1853-1891, we used the median date of 1873 as the sampling date for VK08 in phylogenetic analyses.

Mütter catalogue # 17090.29, a “Vaccination set, made by J.H. Gemrig, Philadelphia,” produced libraries VK12, VK13, and VK14 (Additional File 2: Table S1). J.H. Gemrig, Philadelphia was a company founded in 1839, but the kit was most likely manufactured before 1879. After that year the company became known as “J.H. Gemrig & Sons.” The kit likely dates between 1839 – 1879. The museum database contains no donor or other information. However, according to notes made by the Mütter Museum curator in 1984, the handwritten catalogue entry for the kit was made circa 1933-1937. We used the median year, 1859, of the J.H. Gemrig company as the sampling date for VK12 in time-dependent phylogenetic analyses.

**Detailed Laboratory Materials and Methods**

All procedures were carried out in the clean room laboratory of the McMaster Ancient DNA Centre (Hamilton, Ontario, Canada), which includes physically separate facilities for pre- and post-PCR material.

**Sampling**

The bench tops and non-disposable tools were cleaned with a 6% bleach solution and UV-irradiated double-distilled water between sample manipulations.

*Organic material*

Between 4.3 and 82.2 mg of organic material was crushed using a clean, disposable scalpel blade in a 2.0-mL MAXYmum recovery tube. 0.5 mL of digestion buffer (20 mM Tris-HCl pH 8.0, 5 mM calcium chloride, 50 mM dithiothreitol (DTT), 2.5 mM N-phenacylthiazolium bromide (PTB), 1% polyvinylpyrrolidone (PVPD), 0.5% sarcosyl, 20 mg/mL proteinase K, and nuclease-free water) was added to the sample, which was then incubated for 24 h at 25°C with agitation at 1000 RPM (44). The samples were pelleted via centrifugation at 13,200 RPM for three min, and the supernatant was removed via pipette. The digestion was repeated twice for VK01, VK02, and VK05, resulting in 1 mL of supernatant, and four times for VK08 and VKB01, resulting in 2 mL of supernatant. The samples were extracted using a modified version of the guanidinium method “B” outlined in Glocke and Meyer (2M guanidinium hydrochloride, 70% isopropanol, 0.05% Tween-20) (45).

*Inorganic material – metal*

Metal boxes (such as VK12) and lancets were washed with 0.5 mL of digestion buffer (as above) using a pipette. They were rinsed with 0.5 mL of nuclease-free water, which was combined with the digestion buffer. This solution was used as "the sample". To avoid any potential damage to the items, any lingering digestion buffer was removed by rinsing all items four times with 0.5 mL nuclease free water, gently dried with a Kimwipe, and left to air dry for at least 1 h. The 1 mL of sample was extracted using the modified method “B” from Glocke and Meyer (45), as above. We monitored the items every month post rinse to ensure no damage (i.e. rust) had taken place and have not seen anything as of 24 months post-sampling (Additional File 11: Figure S6).

*Inorganic material – glass*

Materials made of glass (slides) were incubated in 40 mL of extraction buffer from the modified method “B” Glocke and Meyer (45) as above in 50-mL falcon tube, size permitting, or clean antistatic weigh boats covered with ultraclean foil at room temperature. Slides were flipped with clean forceps after 20 h. The slides were removed from the extraction buffer after 24 h and the liquid was saved in a clean 50-mL falcon tube. The slides were placed into 100 mL of nuclease-free water and were agitated and flipped every five min for 20 min. After 20 min, the slides were placed into a fresh 100 mL of nuclease-free water and were agitated and flipped every five min for a second 20 min. The slides were dried carefully with a Kimwipe and left to air dry for 1 h, flipping every 15 min. The 40 mL of extraction buffer was used as input to extraction following Glocke and Meyer (45). A similar protocol using lysis buffer was previously used to recover aDNA from archival glass slides still bearing visible evidence of *Plasmodium* parasites (46).

**Library preparation**

*Single stranded*

Libraries from all extracted materials were prepared following the method in Gansauge et al. (47), using 5 μL extract as template. Sequencing libraries prefaced by an “S” indicate use of single-stranded library preparation.

*Double stranded*

Libraries from samples VK01, VK02, VK05, VK08, VK09, VK12, and blanks were constructed using 5 μL extract as template following Meyer and Kircher (48), with several modifications. Instead of using the suggested SPRI beads for purifications we used the Qiaquick Nucleotide Removal Kit (Qiagen) for purification between blunt-end repair and adapter ligation steps, and a Minelute PCR Purification kit (Qiagen) was used between adapter ligation and end-fill steps. Modifications to both Qiagen kits are as follows: binding and wash steps were done at 6,000 RPM, buffer PE wash was done twice with 650 μL instead of once with 750 μL. Dry spin was done twice at maximum speed for 30 sec with a 180° rotation between spins instead of a single one minute spin at maximum speed, and the elution buffer was incubated on the membrane for 5 min at RT before the elution spin. The adapter ligation incubation was done overnight (for 15 h) at 16°C instead of 30 min at 22°C. The fill-in reaction was inactivated at 80°C for 20 min and was not purified, instead it was taken directly into indexing. Sequencing libraries prefaced by a “D” indicate use of double-stranded library preparation.

**Indexing**

For both single-stranded and double-stranded library preparations, indexing was performed following the strategy described by Kircher, Sawyer, and Meyer (49). Reactions were performed in 40 μL composed of 20 μL KAPA SYBR® FAST qPCR Master Mix (2X), 3 μL of 100 μM forward indexing primer, 3 μL of 100 μM reverse indexing primer, 1.5 μL nuclease-free water, and 12.5 μL of template. For the single-stranded method, libraries were amplified for 10 cycles. For the double-stranded method, the libraries were amplified for 12 cycles. Purification was performed using a Minelute PCR Purification Kit (Qiagen) with the above modifications and eluted in 15 μL buffer EB.

**Targeted enrichment**

*Bait design*

The genomes of 202 OPXV (Additional File 12: Table S5) were compiled, aligned using Mafft v7.205 (40). Gaps were removed from the alignment and strings of 10 or fewer Ns were replaced with Ts. Using local RepeatMasker (50), simple repeats and low-complexity DNA (<1% alignment) were masked. From this masked alignment, 80-mer baits with 3x tiling-density were designed (1,431,875 raw baits), then filtered with USEARCH to remove baits with $\geq$95% identity and 83.125% overlap (decreased to 54,818 baits) (51). The final filtering step was to BLAST baits to the hg38 human genome and mm10 mouse genomes and remove baits with a least one blast hit to either of these genomes. This filtering resulted in a final set of 54,047 unique baits designed and manufactured at Arbor Biosciences (Ann Arbor, MI) as part of a custom myBaits® targeted enrichment kit.

*OPXV targeted enrichment*

- Samples were enriched using baits targeting all available OPXV genomes (Additional File 12: Table S5) according to the protocol in the myBaits v4 manual (Arbor Biosciences), using 10x concentrated Block O to result in the recommended final concentrations, but allowing a maximum of 9.05 μL template input instead of 7 μL template input, using 100 ng of baits per reaction, and using 20 μL of beads (pre- and post-wash) instead of 30 μL pre-wash and 70 μL post-wash. Samples were reamplified with the following conditions: 1X KAPA SYBR ® FAST qPCR Master Mix (2X), 150 nM of each primer (IS5_long_amp.P5 and IS6_ long_amp.P7), and 18.8 µL template. The cycling conditions were: 5 min of initialization at 95˚C, followed by 12 cycles of a 30 second denaturation at 95˚C and a 45 second annealing/extension at 60˚C, followed by a final extension for 3 min at 60°C, and ending with a 1 min hold at 4˚C.

**Quantification, pooling, and sequencing**

Single-stranded libraries were diluted 1 in 1,000 in EBT (10 mM Tris, 0.05% Tween-20) and double-stranded libraries were diluted 1 in 10,000 in EBT for quantification. Reactions composed of 5 μL KAPA SYBR® FAST qPCR Master Mix (2X), 0.2 μL of each primer (100 μM), 0.6 μL nuclease-free water, and 4 μL sample. The reactions were run against the 425–525 base pair PhiX Control (Illumina) standard serially diluted from 0.1 nM to 0.0625 pM, initialized at 95°C for 5 min, and run for 35 cycles under the conditions: 95°C for 30 sec, 60°C for 45 sec.

Samples were pooled at equimolar concentrations for sequencing, except low concentration blanks, for which the maximum volume was pooled. The pooled sample was concentrated over a Minelute PCR Purification kit column (Qiagen), with the modifications above, eluted in 12 μL buffer EB and size selected via gel excision from 150 bp to 500 bp on a 3% NuSieve GTG Agarose Gel, run for 35 minutes at 100 V. The gel slices were purified using a Minelute Gel Extraction kit (Qiagen), with the above modifications, and eluted in 20 μL buffer EB.

Sequencing was performed at the Farncombe Metagenomics Facility at McMaster University. Single-stranded libraries were sequenced on 100% of an Illumina MiSeq v3 run using 2x75 chemistry and using the custom sequencing primer CL72 instead of the standard R1 primer. Double-stranded libraries were sequenced on 4% of an Illumina HiSeq 1500 Rapid v2 run using 2x90 chemistry and using the custom sequencing primer CL72 instead of the standard R1 primer. Double-stranded libraries DVK01, DVK02, DVK08, DVK09, and blanks were sequenced on 47.8% of an additional Illumina HiSeq 1500 Rapid v2 run using 2x90 chemistry using the standard R1 primer. Double-stranded enriched libraries and blanks were sequenced on 4.75% of an Illumina HiSeq 1500 Rapid v2 run using the standard R1 primer. Data generated from the MiSeq run was processed with the MiSeq Reporter software and data from the HiSeq runs were processed with bcl2fastq(52).

**Detailed Computational Analysis**

**Bioinformatic Processing**

Demultiplexed reads were trimmed of either the ssLP and dsLP adapters and merged using ancient DNA appropriate parameters (--ancientdna) with the program leeHom v1.1.5 (53). Trimmed and merged reads were mapped to a reference of interest, either VACV strain Copenhagen (M35027), VARV strain India-1967 (NC_001611), the revised Cambridge Reference Sequence (NC_012920) or the hard-masked version human genome hg38 (http://hgdownload.soe.ucsc.edu/goldenPath/hg38/bigZips/), using a modified version of BWA (<https://github.com/mpieva/network-aware-bwa>) (36) with a maximum allowable edit distance of 0.01 (-n 0.01), maximum two gap openings (-o 2) and without seeding (-l 16500). For a given reference of interest, mapped reads were further filtered to those that were merged or single and properly paired (<https://github.com/grenaud/libbam>), with unique 5’ and 3’ mapping coordinates (<https://bitbucket.org/ustenzel/biohazard>). Mapped reads were further filtered to those a minimum of 35 bp in length and with a mapping quality of at least 30 for VACV references and restricted to minimum of 24 bp and map quality of at least 30 for the hg38 and rCRS references.

**Ancient DNA Authentication**

Libraries were first assessed to evaluate that fragment length distributions were short as would be expected for authentic ancient damaged DNA. When mapped to both VACV and the human rCRS references, average fragment lengths for shotgun the libraries ranged between 40-52 bp with an average of 6-10 bp increase observed following enrichment (Additional File 2: Table S1, Additional File 4: Table S2). Additionally, we examined DNA reads for evidence of hydrolytic damage and deamination through mapDamage 2.0 (54), which showed consistent though low evidence of terminal deamination as might be expected for samples that are <200 years old (Additional File 3: Figure S1, Additional File 13: Figure S7) (55).

**Metagenomic Profiles**

Profiles of all molecules >=35bp from the sequenced shotgun libraries were assessed by BLAST v2.6.9 (56) using the megablast function and retaining only hits with an e-value of <= 0.001 against a local installation of the nucleotide database updated on March 24, 2017 and further visualised with KronaTools 2.7 (24). Blast and Krona analyses indicate that molecules of human origin dominated the compositions of VK01, VK02 and VK08 with VACV components ranging between 0.3% and 2.0%. Notably, there was very little indication of additional bacterial components, with no indication of *Treponema,* and only 0.3% of reads matching to *Staphylococcus aureus* in the VK02 shotgun profile. Major components of the Krona profiles were simplified and reproduced through the R package ggplot2 (25) (Figure 1B&C). To exclude contamination from modern *S. aureus* material, reads were compared to the Comprehensive Antibiotic Resistance Database (57) using Bowtie2 (58) and BLASTX, including both CARD’s canonical sequences and *in silico* prediction of AMR alleles (CARD’s Resistome & Variants data) (CARD 3.0.0, Resistomes 3.0.2). Additionally, reads were compared to a custom set of diagnostic *k*-mers designed from CARD data for identification of species-of-origin for predicted AMR nucleotide sequences (Additional File 14: Table S6).

***De novo* Assembly**

We reassembled the viral genome of the VK01 strain using SPAdes v 3.6.1 (59), wherein reads merged by leeHom were treated as single-end sequences and those trimmed but unmerged as paired-end sequences (53). To minimise the effects of short fragment lengths and repetitive regions, we restricted input reads to those at least 50 bp in length. To avoid any premature capping of contigs by residual adapter sequences or chimeras, any reads with an edit distance of 4 or less to any of the ssLP or dsLP library adapters were removed. This resulted in a contig 184,677 bp in length. The SPAdes assembly for VK01, as well as the less successful assemblies for VK02 and VK08 were evaluated with QUAST v3.1, summary statistics of the *de novo* assemblies are summarised in Additional File 5: Table S3.

**Consensus VACV genomes**

The remaining samples did not have the shotgun sequencing read length and depth to generate *de novo* viral genomes. Following the addition of targeted enrichment data, consensus sequences from VK02, VK05, VK08 and VK12 were called by first mapping the reads to the VK01 *de novo* assembly (as described above) and then using the endoCaller function of schmutzi v1.0 to generate a consensus sequence with a high quality threshold (-qual 60) (37). We confirmed that the consensus sequence was restricted to positions with at least 10x coverage and 0.9 variant frequency by mapping the raw reads back to the schmutzi-generated consensus sequence and manually curating any positions that did not meet the pre-defined coverage and allele frequency thresholds. This process was further repeated with three additional VACV references genomes (Copenhagen strain M35027, Western Reserve strain AY243312, and Dryvax strain JN654986 to confirm that the generated consensus sequences were unbiased by the reference (Additional File 6: Figure S2).

**Phylogenetic Analyses**

To derive a data set that was appropriate for phylogenetic analysis we first aligned the Mütter Museum vaccination kit strains with 202 publicly available OPXV genomes and then inferred preliminary maximum likelihood trees on these data. Over several iterations, it was clear that we could reduce the number of comparative OPXV sequences used for our phylogenetic analysis to 76 representatives, including the Mulford’s 1902 vaccine strain (6), and root the tree with three strains of ectromelia virus (ECTV) that obviously represented outgroup taxa (Figure 2 and Additional File 9: Table S4). The number of MPXV and VARV strains included in the phylogeny was restricted as both viruses formed securely positioned clades. The remaining 81 OPXV genomes were aligned using MAFFT v7.205 (40) to a final length of 263,227 bp. The initial alignment was then processed through Gblocks 0.91b (41) to remove poorly aligned regions and indels producing a final alignment of 134,607 bp. The phylogenetic relationship among these OPXVs was inferred using the maximum likelihood method available in PhyML (42), employing the GTR model of nucleotide substitution with four discrete gamma categories (Figure 2). The robustness of the resulting inferred tree was assessed through bootstrap analysis with 1000 replicates. To ensure that our mapping reference genome was not affecting phylogenetic positioning of our ancient strains, we repeated the entire process by mapping to additional VACV genome references - the Copenhagen, Western Reserve and Dryvax genomes - and generating alignments for the five Mütter vaccination kit strains (Additional File 4: Figure S2).

We assessed the extent of clock-like structure within both the OPXV alignment and a reduced alignment of 30 VACV-like strains by calculating the regression of root-to-tip distances against their year of sampling or sequencing using the linear model function available through R (60). In iterations using best-approximate sampling date for the available genomes, as well as randomised tip dates, we found no evidence for a consistent relationship between sampling or sequencing time (year) and root-to-tip genetic distance in the ML phylogenies. Accordingly, we conclude that there is no support for a molecular clock (i.e. rate constancy) in these data such that we cannot use them to estimate divergence times (Additional File 8: Figure S4).

Metadata, including the virus strain, country of origin and host of isolation were superimposed on the maximum likelihood tree using GrapeTree v1.5.0 (61) (Additional File 7: Figure S3). We tested for possible recombination within tree topologies by splitting the 134,607 bp alignments of 81 orthopoxvirus strains into four consecutive regions ~33,652 bp each. Four phylogenetic trees were then inferred on these regions using PhyML (43), employing the GTR model based on analysis from Modeltest-ng 2017 release (42). Subsequently, the robustness of each tree was assessed using a bootstrap analysis with 1000 replicates (Additional File 10: Figure S5).

The bias associated with targeted enrichment, in that baits can only be designed for sequences that already exist in publicly available databases and not all sequences are recovered with equal probability, makes enrichment data poor input for *de novo* assembly algorithms. The bias in recovery of VK01 data against three reference genomes is evident through Circos(62) analysis of coverage depth and GC content (Additional File 15: Figures S8-10). While we do not recommend this strategy, nevertheless, we attempted phylogenetic analysis of the *de novo* assemblies produced using enriched data following the same protocol as above. The phylogenetic positioning of the Mütter strains is consistent when using *de novo* assemblies generated from the product of targeted enrichment (Additional File 16: Figure S11) following the same protocol as above. However, we note that while the individual assemblies may have been longer than those produced with shotgun data only (Additional File 5: Table S3), the ultimate Gblocked alignment was considerably shorter in this phylogeny (95,420 bp retained of the original 264,669 bp alignment) (Additional File 16: Figure S11).

Figure S1: Terminal damage rates for VK01, VK02, VK05, VK08 and VK12 libraries when mapped to either VACV strain Copenhagen reference (M35027) or human mitochondrial reference (rCRS).

Figure S2: Maximum likelihood trees for Mutter vaccine strains (indicated in red) in which consensus sequences were called in reference to alternative VACV genomes (indicated in blue). Nodes with bootstrap values > 95% are indicated with asterisks. All horizontal branch lengths are scaled according to the number of nucleotide substitutions per site. (A) VK02, VK05, VK08 and VK12 consensus sequences called in reference to VK01 *de novo* assembly contig; (B) VK01, VK02, VK05, VK08 and VK12 consensus sequences called in reference to Copenhagen strain (M35027); (C) VK01, VK02, VK05, VK08 and VK12 consensus sequences called in reference to Western Reserve strain (AY243312); (D) VK01, VK02, VK05, VK08 and VK12 consensus sequences called in reference to Dryvax strain (JN654986).

Figure S3: Maximum likelihood tree of 79 OPXV with metadata included. (A) 79 OPXV assigned to viral strain; (B) 79 OPXV coloured by country of sample origin; (C) 79 OPXV coloured by host of isolation.

Figure S4: Regression analyses depicting the relationship between root-to-tip genetic distance on the ML phylogeny against either year of collection or year of sequencing, both showing an absence of molecular clock. (A) 73 OPXV genomes dated to best the approximation of strain collection; (B) 81 OPXV genomes dated to year of genome sequencing; (C) 23 VACV genomes dated to the best approximation of strain collection; (D) 30 VACV genomes dated to year of genome sequencing. Also see Additional File 9: Table S4.

Figure S5: Partitioned maximum likelihood analysis used to assess the extent of recombination in these poxviruses. Nodes with > 95% bootstrap support are indicated by asterisks. All horizontal branch lengths are scaled according to the number of nucleotide substitutions per site. Each phylogenetic tree was estimated from ~33,652 bp using the Gblocked 81 OPXV alignment. Respectively, each panel refers to four sections of the alignment: (A) 1-33,652 bp; (B) 33,653-67,304 bp; (C) 67,305-100,956 bp; (D) 100,957-134,607 bp.

Figure S6: Mütter catalogue # 17090.29. Left panel: before non-destructive sampling. Right panel: 14 months after non-destructive sampling of the glass slides, metal box and lancet blades.

Figure S7: Comparison of terminal damage patterns for VK01 library mapped to VACV Copenhagen (M35027) reference under different library conditions. A) single-stranded library prep B) double-stranded library prep C) double-stranded library prep with 10x adapters (2.5 μM concentration) D) double-stranded library prep with 10x adapters and reduced (0.1x) library input for indexing. All libraries were shotgun sequenced.

Figure S8: VK01 coverage relative to Copenhagen VACV strain M35027. From interior to exterior: the solid purple band represents the Copenhagen reference genome, the green band represents the coverage of the OPXV baits relative to the Copenhagen reference. The pale purple band indicates the GC content of the Copenhagen reference genome in 50 bp windows, while the black line indicates the genomic average of 0.33 GC content. The outer purple band indicates the gene annotations of the Copenhagen M35027 genome. The outermost red band indicates the relative coverage depth of the VK01 sample to the Copenhagen reference in 50bp windows. The concentric grey lines indicate intervals of 250x coverage.

Figure S9: VK01 coverage relative to GRI90 CPXV strain X93455. From interior to exterior: the solid yellow band represents the GRI90 reference genome, the green band represents the coverage of the OPXV baits relative to the GRI90 reference. The pale yellow band indicates the GC content of the GRI90 reference genome in 50 bp windows, while the black line indicates the genomic average of 0.34 GC content. The outer light yellow band indicates the gene annotations of the GRI90 X93455 genome. The outermost red band indicates the relative coverage depth of the VK01 sample to the GRI90 reference in 50bp windows. The concentric grey lines indicate intervals of 250x coverage.

Figure S10: VK01 coverage relative to Horsepox strain DQ792504. From interior to exterior: the solid purple band represents the Horsepox reference genome, the green band represents the coverage of the OPXV baits relative to the Horsepox reference. The pale purple band indicates the GC content of the Horsepox reference genome in 50 bp windows, while the black line indicates the genomic average of 0.33 GC content. The outer purple band indicates the gene annotations of the Horsepox DQ792504 genome. The outermost red band indicates the relative coverage depth of the VK01 sample to the Horsepox reference in 50bp windows. The concentric grey lines indicate intervals of 250x coverage.

Figure S11: Maximum likelihood phylogenetic analysis of historical vaccine strains in relation to other OPXV using largest de novo assembled contig for each sample. Produced using Gblocked alignment of 95,420 positions (36% of original alignment).

Table S1: Information on the libraries generated from the Mütter Museum vaccination kits and vaccinia virus mapping statistics.

Table S2: Mapping statistics for human component of libraries VK01, VK02 and VK08.

Table S3: Summary statistics produced by QUAST for SPAdes *de novo* assemblies of VK01, VK02 and VK08 from shotgun data filtered for edit distance of 4 from all library preparation adapters and restricted to molecules at either a minimum of 30, 40, 50 or 60bp.

Table S4: OPXV genomes used for comparison in the phylogenetic analyses.

Table S5: OPXV genomes used for bait design.

Table S6: BLASTX, Bowtie2, and taxonomic *k*-mer annotation of VK02 reads against CARD reference sequences.

**References**

1. Castro R, Casanas B. Orthopoxviruses and Human Disease. In: Global Virology II-HIV and NeuroAIDS. Springer; 2017. p. 689–97.

2. Fenner F, Henderson DA, Arita I, Jezek Z, Ladnyi ID. Smallpox and its eradication. Vol. 6. World Health Organization Geneva; 1988.

3. Hopkins DR. The greatest killer: smallpox in history. University of Chicago Press; 2002.

4. Jenner E. An Inquiry into the Causes and Effects of the Variolae Vaccinae: A Disease Discovered in Some of the Western Counties of England, Particularly Gloucestershire, and Known by the Name of Cow Pox. London: Printed for the author by Samson Low, No. 7, Berwick Street, Soho.; 1798.

5. Esparza J, Schrick L, Damaso CR, Nitsche A. Equination (inoculation of horsepox): An early alternative to vaccination (inoculation of cowpox) and the potential role of horsepox virus in the origin of the smallpox vaccine. Vaccine. 2017; 35(52):7222-7230.

6. Schrick L, Tausch SH, Dabrowski PW, Damaso CR, Esparza J, Nitsche A. An Early American Smallpox Vaccine Based on Horsepox. New England Journal of Medicine. 2017;377(15):1491–1492.

7. Martin HA. Report on Animal Vaccination. Trans Am Med Assoc. 1877;XXVII:187–248.

8. Funkhouser WK. Edward Jenner and vaccination: The road to elimination of epidemic smallpox. American Society for Investigative Pathology. 2016;1.

9. Baxby D, Gaskell C, Gaskell R, Bennett M. Ecology of orthopoxviruses and use of recombinant vaccinia vaccines. The Lancet. 1986;328(8511):850–1.

10. Baxby D, Bennett M, Getty B. Human cowpox 1969–93: a review based on 54 cases. Br J Dermatol. 1994;131(5):598–607.

11. Esparza J. Has horsepox become extinct? Veterinary Record. 2013;173(11):272–3.

12. Downie AW. A study of the lesions produced experimentally by cowpox virus. Journal of Pathology and Bacteriology. 1939;48:361–79.

13. Downie AW. Immunological relationship of the virus of spontaneous cowpox to vaccinia virus. British Journal of Experimental Pathology. 1939;20:158–76.

14. Baxby D. Poxvirus hosts and reservoirs. Archives of Virology. 1977;55(3):169–79.

15. Baxby D. The origins of vaccinia virus. Journal of Infectious Disease. 1977;136(3):453–5.

16. Qin L, Upton C, Hazes B, Evans DH. Genomic analysis of the vaccinia virus strain variants found in Dryvax vaccine. Journal of Virology. 2011;85(24):13049-13060.

17. Qin L, Favis N, Famulski J, Evans DH. Evolution of and evolutionary relationships between extant vaccinia virus strains. Journal of Virology. 2015;89(3):1809–24.

18. Sánchez-Sampedro L, Perdiguero B, Mejías-Pérez E, García-Arriaza J, Di Pilato M, Esteban M. The evolution of poxvirus vaccines. Viruses. 2015;7(4):1726–803.

19. Baxby D. Jenner’s smallpox vaccine. The Riddle of the Origin of Vaccinia Virus. Heinemann Lond. 1981;12:997–1035.

20. Harris E. Vaccination in the Army. In: Flint A, editor. Contributions Relating to the Causation and Prevention of Disease. New York: US Sanitary Commission/Hurd and Houghton; 1867.

21. United States Durgeon-General’s Office. In: The Medical and Surgical History of the War of Rebellion. Washington, D.C. US Government Printing Office. 1888. Available from: http://resource.nlm.nih.gov/14121350R.

22. Jones J. Researches Upon "Spurious Vaccination,": Or the Abnormal Phenomena Accompanying and Following Vaccination in the Confederate Army During the Recent American Civil War, 1861-1865. Vol. 1376. University Medical Press; 1867.

23. Hicks R. Scabrous Matters: Spurious Vaccination in the Confederacy. In: Cashin J, editor. War Matters: Material Culture of the Civil War Era. University of North Carolina Press; 2018.

24. Ondov BD, Bergman NH, Phillippy AM. Interactive metagenomic visualization in a Web browser. BMC Bioinformatics. 2011;12(1):385.

25. Wickham H. ggplot2: Elegant Graphics for Data Analysis. New York: Springer-Verlag; 2016. Available from: http://ggplot2.org

26. Skoglund P, Storå J, Götherström A, Jakobsson M. Accurate sex identification of ancient human remains using DNA shotgun sequencing. Journal of Archaeological Sciences. 2013;40(12):4477–82.

27. Devault AM, Mortimer TD, Kitchen A, Kiesewetter H, Enk JM, Golding GB, Southon J, Kuch M, Duggan AT, Aylward W, Gardner SN, Allen JE, King AM, Wright G, Kuroda M, Kato K, Briggs DEG, Fornaciari G, Holmes EC, Poinar HN, Pepperell CS. A molecular portrait of maternal sepsis from Byzantine Troy. Elife. 2017;6:e20983.

28. Tulman E, Delhon G, Afonso C, Lu Z, Zsak L, Sandybaev N, Kerembekova UZ, Zaitsec VL, Kutish GF, Rock DL. Genome of horsepox virus. Journal of Virology. 2006;80(18):9244–58.

29. Alcamí A, Smith GL. Receptors for gamma-interferon encoded by poxviruses: implications for the unknown origin of vaccinia virus. Trends in Microbiology. 1996;4(8):321–6.

30. Gubser C, Hue S, Kellam P, Smith GL. Poxvirus genomes: a phylogenetic analysis. Journal of General Virology. 2004;85(1):105–17.

31. Damaso CR, Esposito JJ, Condit RC, Moussatché N. An emergent poxvirus from humans and cattle in Rio de Janeiro State: Cantagalo virus may derive from Brazilian smallpox vaccine. Virology. 2000;277(2):439–49.

32. Medaglia MLG, Moussatché N, Nitsche A, Dabrowski PW, Li Y, Damon IK, Lucas CG, Arruda LB and Damaso CR. Genomic analysis, phenotype, and virulence of the historical Brazilian smallpox vaccine strain IOC: implications for the origins and evolutionary relationships of vaccinia virus. Journal of Virology. 2015;89(23):11909-11925.

33. Damaso CR. Revisiting Jenner’s mysteries, the role of the Beaugency lymph in the evolutionary path of ancient smallpox vaccines. Lancet Infectious Disease. 2017;18(2):e55–63.

34. Kondas AV, Olson VA, Li Y, Abel J, Laker M, Rose L, Wilkins K, Turner J, Kline R and Damon IK. Variola virus specific diagnostic assays: characterization, sensitivity, and specificity. Journal of Clinical Microbiology. 2015;53(4):1406-1410.

35. Li Y, Olson VA, Laue T, Laker MT, Damon IK. Detection of monkeypox virus with real-time PCR assays. Journal of Clinical Virology. 2006;36(3):194–203.

36. Li H, Durbin R. Fast and accurate short read alignment with Burrows–Wheeler transform. Bioinformatics. 2009;25(14):1754–60.

37. Renaud G, Slon V, Duggan AT, Kelso J. Schmutzi: estimation of contamination and endogenous mitochondrial consensus calling for ancient DNA. Genome Biology. 2015;16(1):224.

38. van Oven M. PhyloTree Build 17: Growing the human mitochondrial DNA tree. Forensic Science International: Genetics Supplement Series. 2015;5:e392–4.

39. Weissensteiner H, Pacher D, Kloss-Brandstätter A, Forer L, Specht G, Bandelt H-J, Kronenberg F, Salas A, and Schönherr. HaploGrep 2: mitochondrial haplogroup classification in the era of high-throughput sequencing. Nucleic Acids Research. 2016;44(W1):W58–63.

40. Katoh K, Standley DM. MAFFT multiple sequence alignment software version 7: improvements in performance and usability. Molecular Biology and Evolution. 2013;30(4):772–80.

41. Talavera G, Castresana J. Improvement of phylogenies after removing divergent and ambiguously aligned blocks from protein sequence alignments. Systematic Biology. 2007;56(4):564–77.

42. Guindon S, Dufayard J-F, Lefort V, Anisimova M, Hordijk W, Gascuel O. New algorithms and methods to estimate maximum-likelihood phylogenies: assessing the performance of PhyML 3.0. Systematics Biology. 2010;59(3):307–21.

43. Zhou Z, Alikhan N-F, Sergeant MJ, Luhmann N, Vaz C, Francisco AP, Carriço JA and Achtman M. GrapeTree: visualization of core genomic relationships among 100,000 bacterial pathogens. Genome Research. 2018;28(9):1395–404.

44. Schwarz C, Debruyne R, Kuch M, McNally E, Schwarcz H, Aubrey AD, Bada J, and Poinar H. New insights from old bones: DNA preservation and degradation in permafrost preserved mammoth remains. Nucleic Acids Research. 2009;37(10):3215–29.

45. Glocke I, Meyer M. Extending the spectrum of DNA sequences retrieved from ancient bones and teeth. Genome Research. 2017 Jul;27(7):1230–7.

46. Gelabert P, Sandoval-Velasco M, Olalde I, Fregel R, Rieux A, Escosa R, Aranda C, Paajimans K, Mueller I, Gilbert MTP, and Lalueza-Fox C. Mitochondrial DNA from the eradicated European Plasmodium vivax and P. falciparum from 70-year-old slides from the Ebro Delta in Spain. Proceedings of the National Academy of Sciences. 2016;113(41):11495–500.

47. Gansauge M-T, Gerber T, Glocke I, Korlević P, Lippik L, Nagel S, Riehl LM, Schmidt A and Meyer M. Single-stranded DNA library preparation from highly degraded DNA using T4 DNA ligase. Nucleic Acids Research. 2017;45(10):e79–e79.

48. Meyer M, Kircher M. Illumina sequencing library preparation for highly multiplexed target capture and sequencing. Cold Spring Harbor Protocols. 2010;2010(6):pdb. prot5448.

49. Kircher M, Sawyer S, Meyer M. Double indexing overcomes inaccuracies in multiplex sequencing on the Illumina platform. Nucleic Acids Research. 2011;40(1):e3–e3.

50. Smit A, Hubley R, Green P. RepeatMasker Open-4.0. 2013–2015. http://www.repeatmasker.org/.

51. Edgar, RC (2010) Search and clustering orders of magnitude faster than BLAST, Bioinformatics 26(19), 2460-2461. https://doi.org/10.1093/bioinformatics/btq461.

52. Duggan AT, Klunk J, Porter AF, Dhody AN, Hicks R, Smith GL, et al. Vaccinia virus Genome sequencing and assembly, Aug 20 ’19. Datasets. Sequence Read Archive. 2019. Available from: https://www.ncbi.nlm.nih.gov/sra/PRJNA561155

53. Renaud G, Stenzel U, Kelso J. leeHom: adaptor trimming and merging for Illumina sequencing reads. Nucleic Acids Research. 2014;42(18):e141–e141.

54. Jónsson H, Ginolhac A, Schubert M, Johnson PL, Orlando L. mapDamage2.0: fast approximate Bayesian estimates of ancient DNA damage parameters. Bioinformatics. 2013;29(13):1682–4.

55. Sawyer S, Krause J, Guschanski K, Savolainen V, Pääbo S. Temporal Patterns of Nucleotide Misincorporations and DNA Fragmentation in Ancient DNA. PLoS ONE. 2012;7(3):e34131.

56. Altschul SF, Gish W, Miller W, Myers EW, Lipman DJ. Basic local alignment search tool. Journal of Molecular Biology. 1990;215(3):403–10.

57. Jia B, Raphenya AR, Alcock B, Waglechner N, Guo P, Tsang KK, Lago BA, Dave BM, Pereira S, Sharma AN, Doshi S, Courtot M, Lo R, Williams LE, Frye JG, Elsayegh T, Sardar D, Westman EL, Pawlowski AC, Johnson TA, Brinkman FSL, Wright GD, McArthur AG. CARD 2017: expansion and model-centric curation of the comprehensive antibiotic resistance database. Nucleic Acids Research. 2016;45(D1):D566–73.

58. Langmead B, Salzberg SL. Fast gapped-read alignment with Bowtie 2. Nature Methods. 2012 Apr;9(4):357–9.

59. Bankevich A, Nurk S, Antipov D, Gurevich AA, Dvorkin M, Kulikov AS, et al. SPAdes: a new genome assembly algorithm and its applications to single-cell sequencing. Journal of Computational Biology. 2012;19(5):455–77.

60. Krzywinski M, Schein J, Birol I, Connors J, Gascoyne R, Horsman D, et al. Circos: an information aesthetic for comparative genomics. Genome Research. 2009;19(9):1639–45.

61. Team RC. R: A language and environment for statistical computing. 2013.

62. Posada D. jModelTest: phylogenetic model averaging. Molecular Biology and Evolution. 2008;25(7):1253–6.
