# Supplementary figures and images for: The origins and genomic diversity of American Civil War Era smallpox vaccine strains
Source: Genome Biol. 2020 Jul 20;21:175. doi: 10.1186/s13059-020-02079-z (PMC7370420; doi:10.1186/s13059-020-02079-z)

VACV

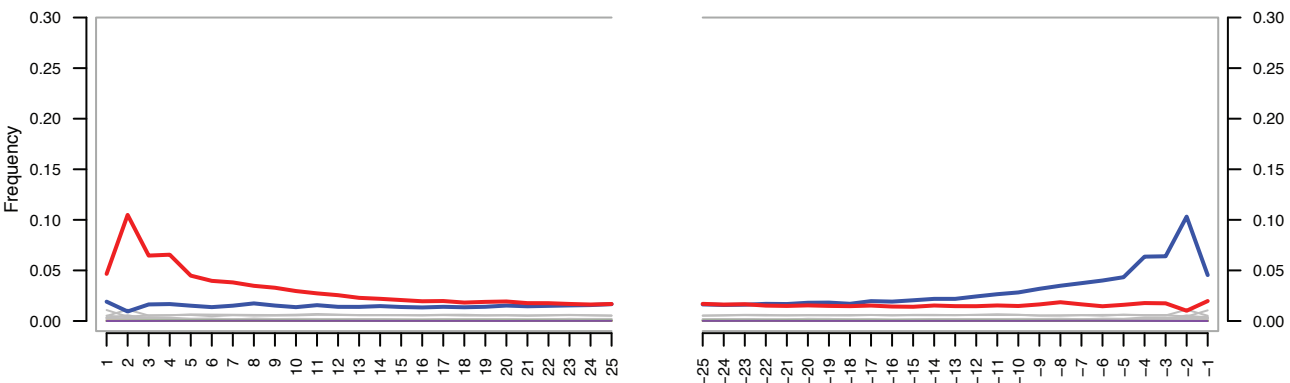

rCRS

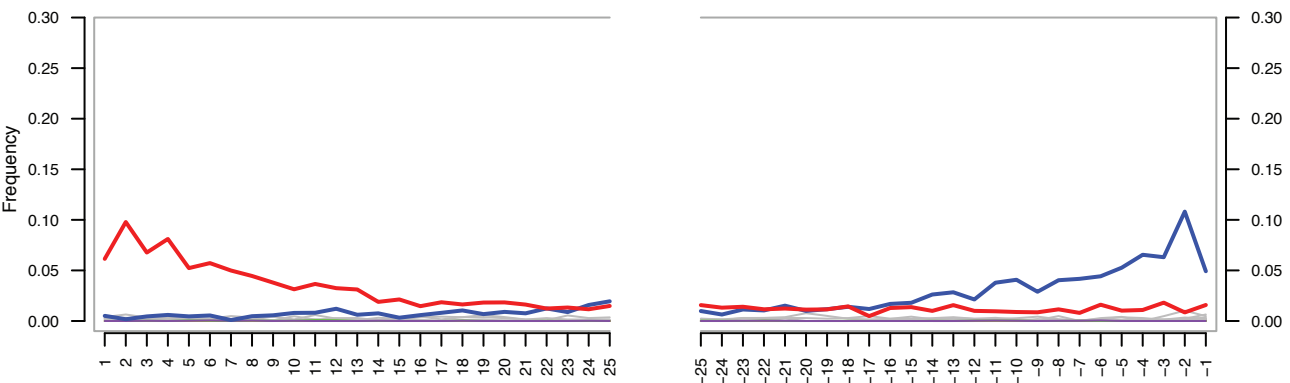

VK01

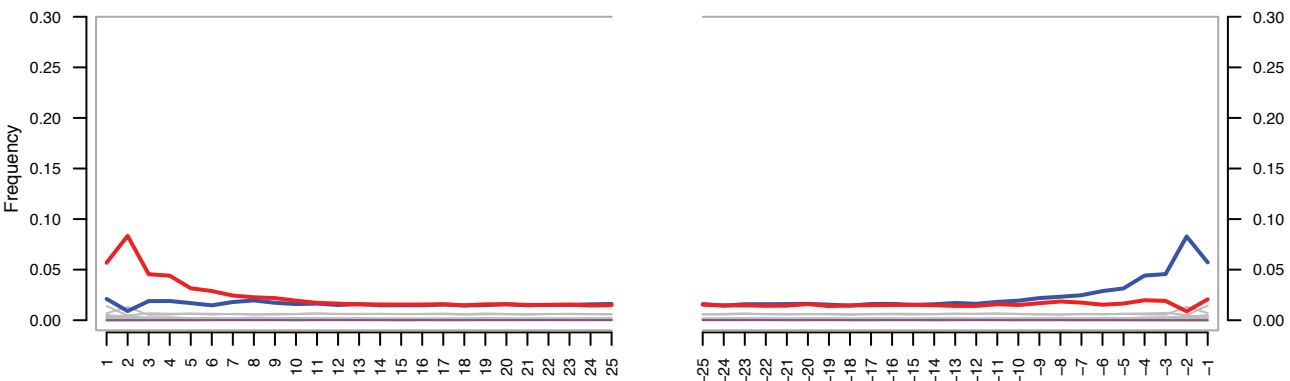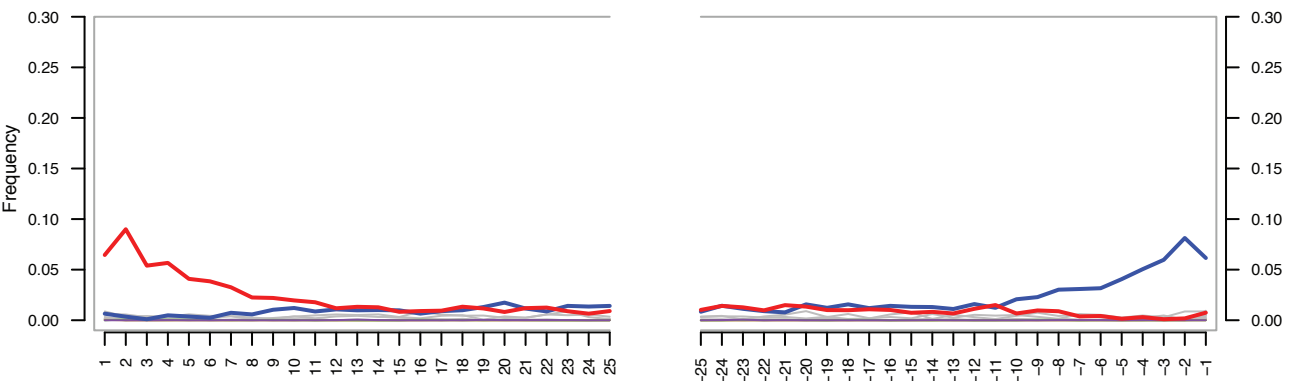

VK02

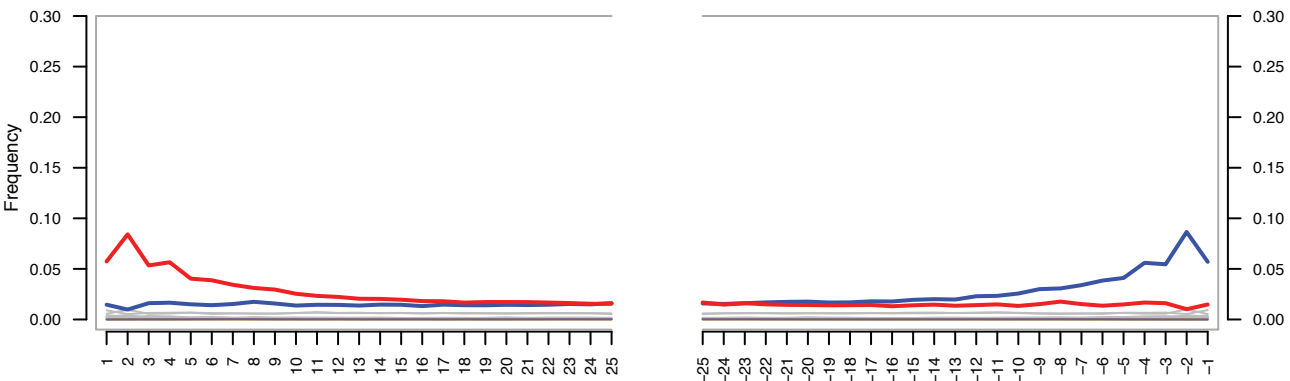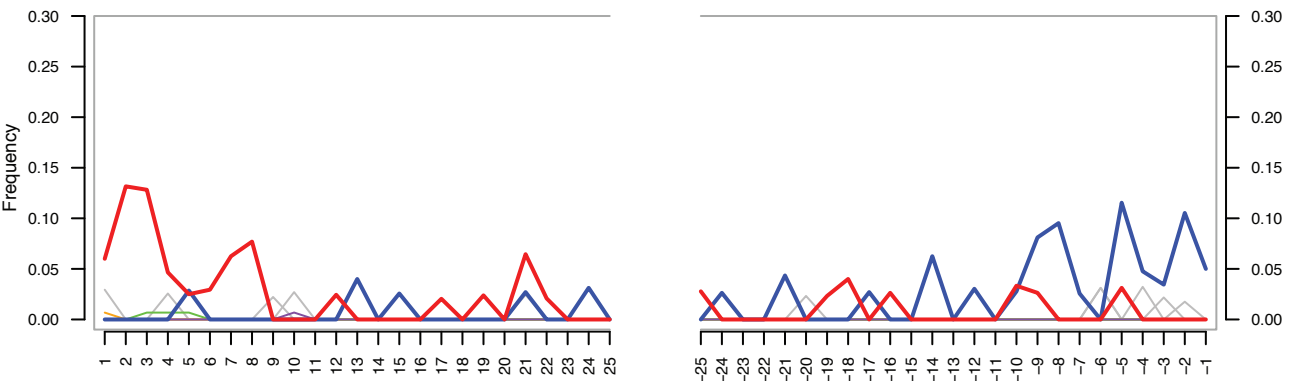

VK05

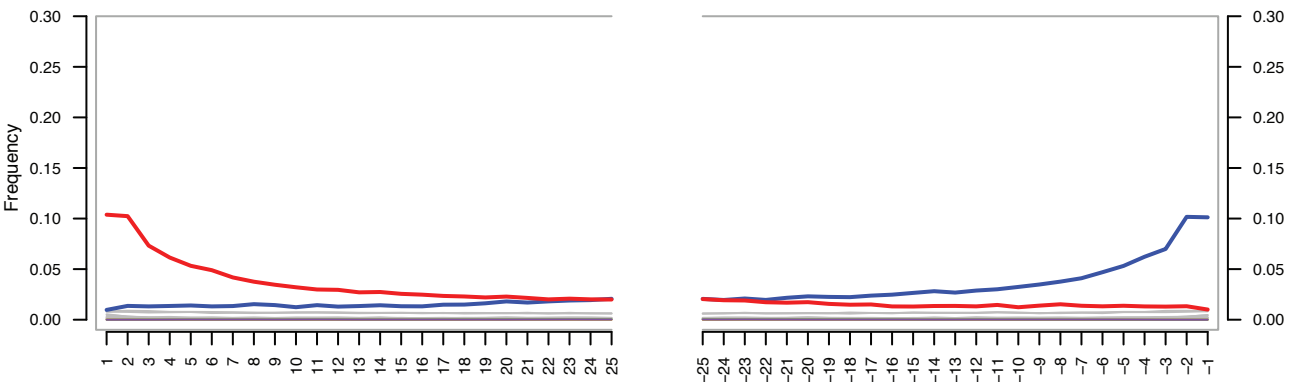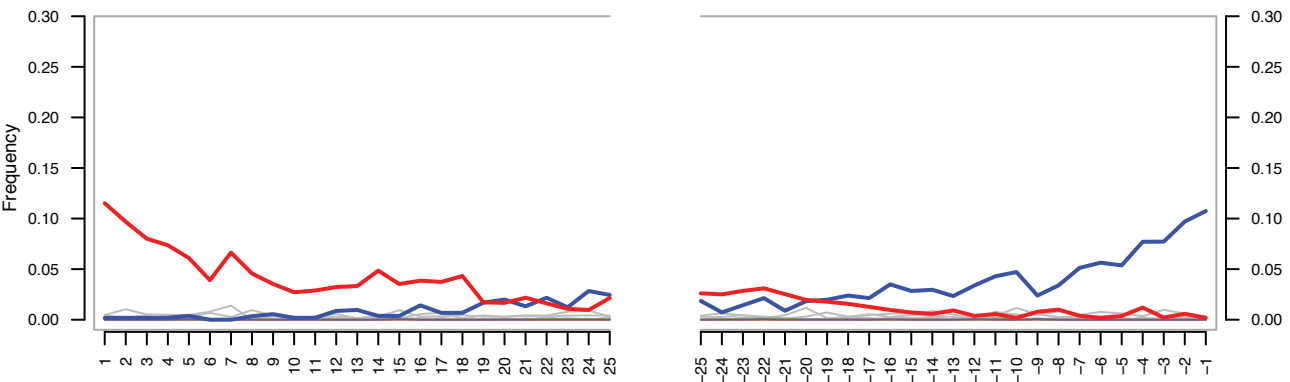

VK08

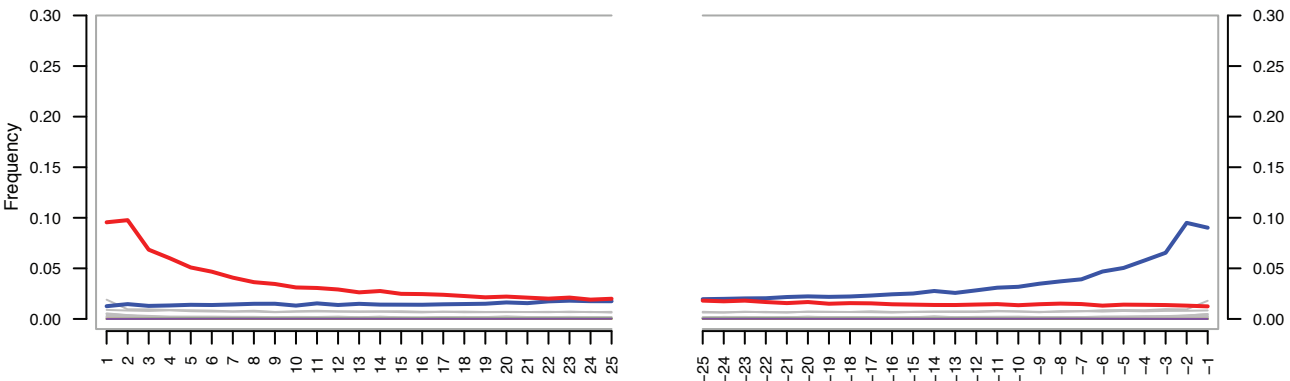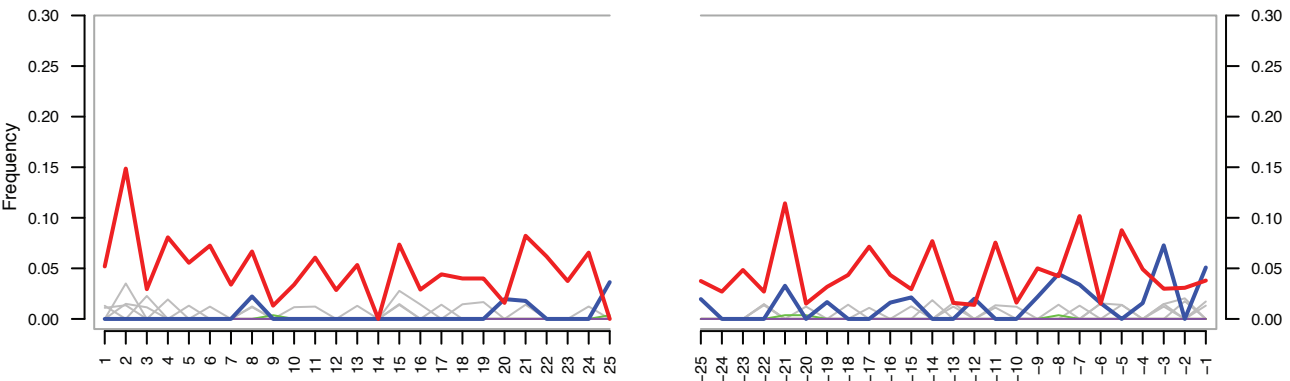

VK12

Supplement: Supplementary file 3 — Additional file 3: Figure S1. Terminal damage rates for VK01, VK02, VK05, VK08 and VK12 libraries mapped to VACV strain Copenhagen reference (M35027) and human mitochondrial reference (rCRS). [file 13059_2020_2079_MOESM3_ESM.pdf]

A

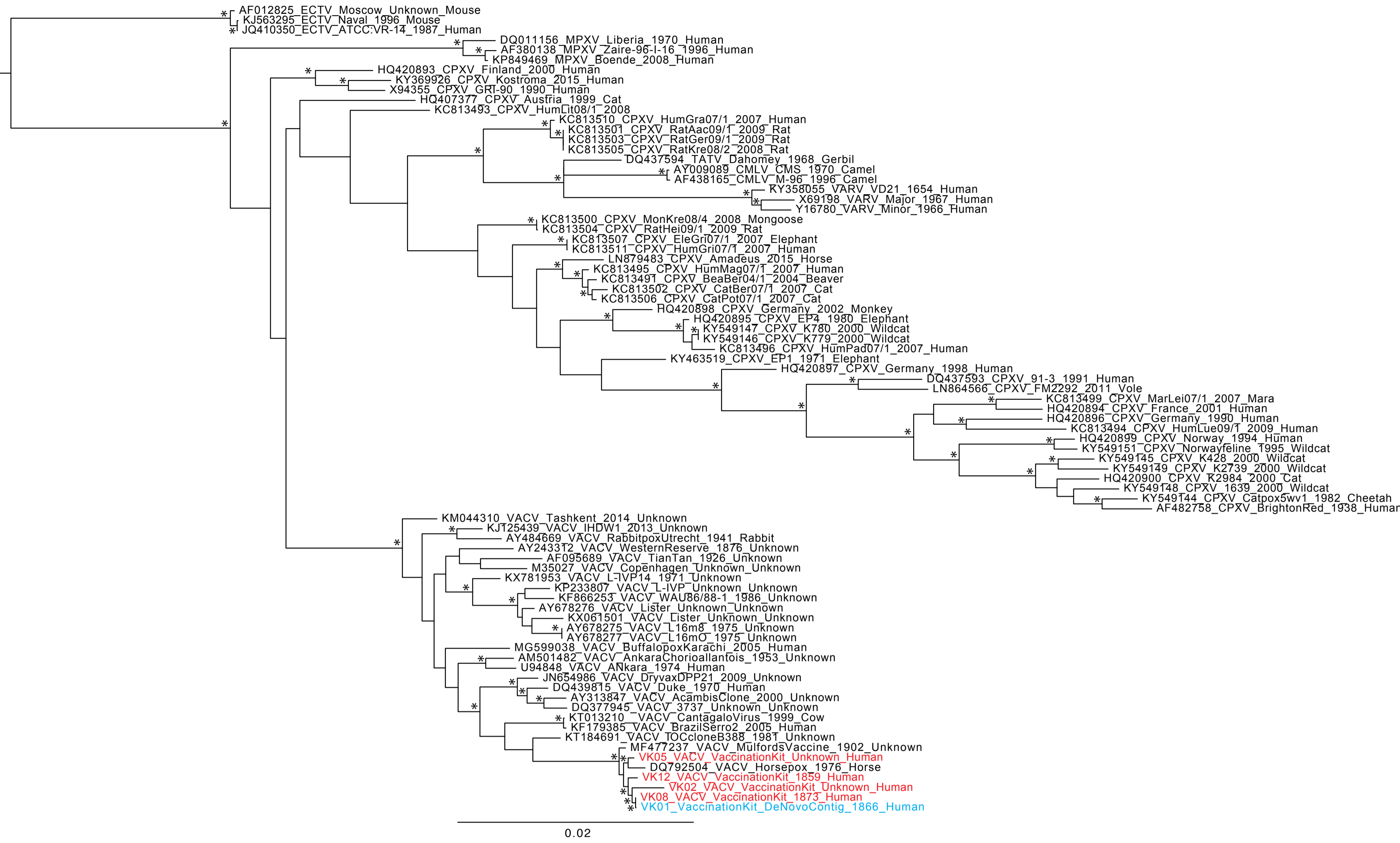

B

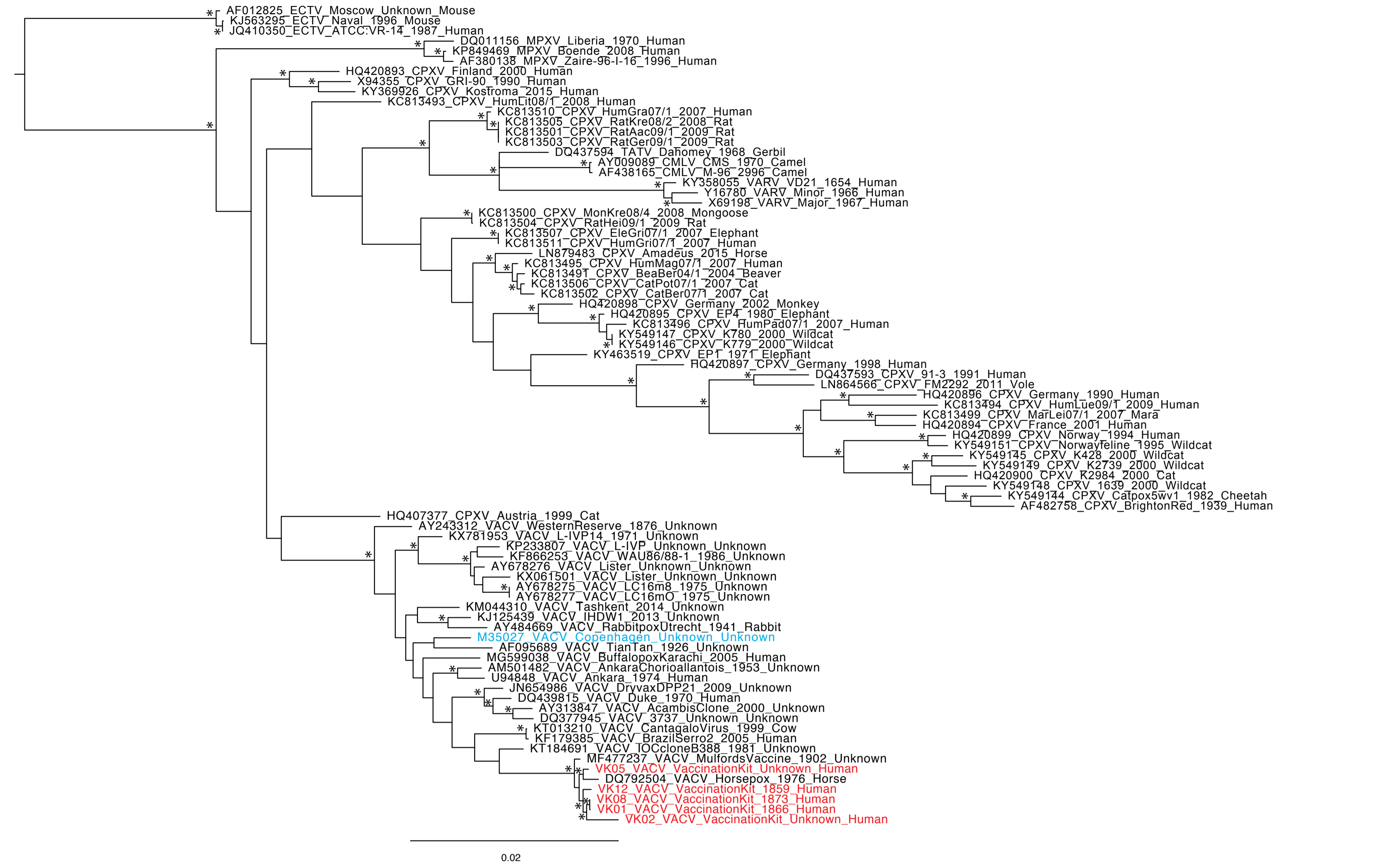

C

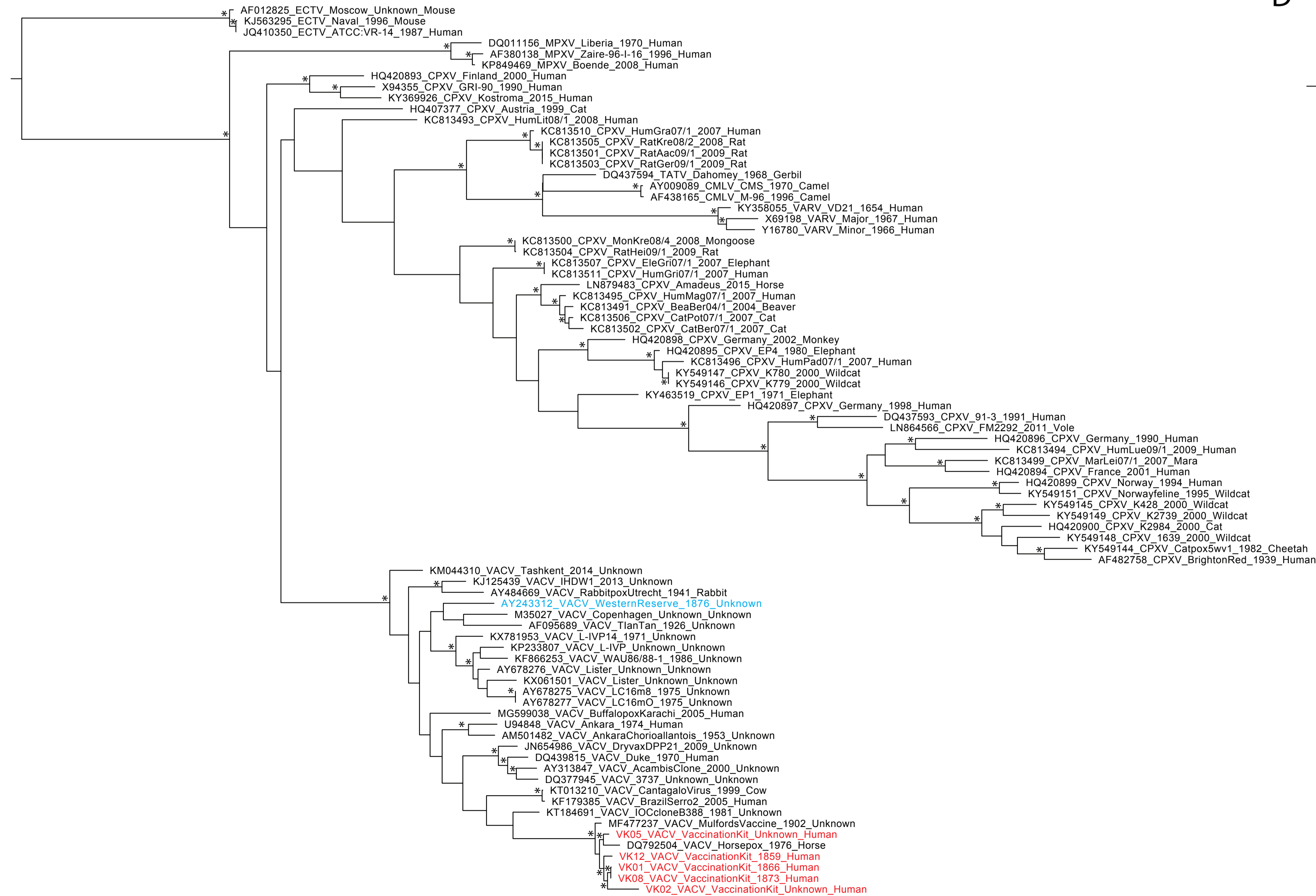

D

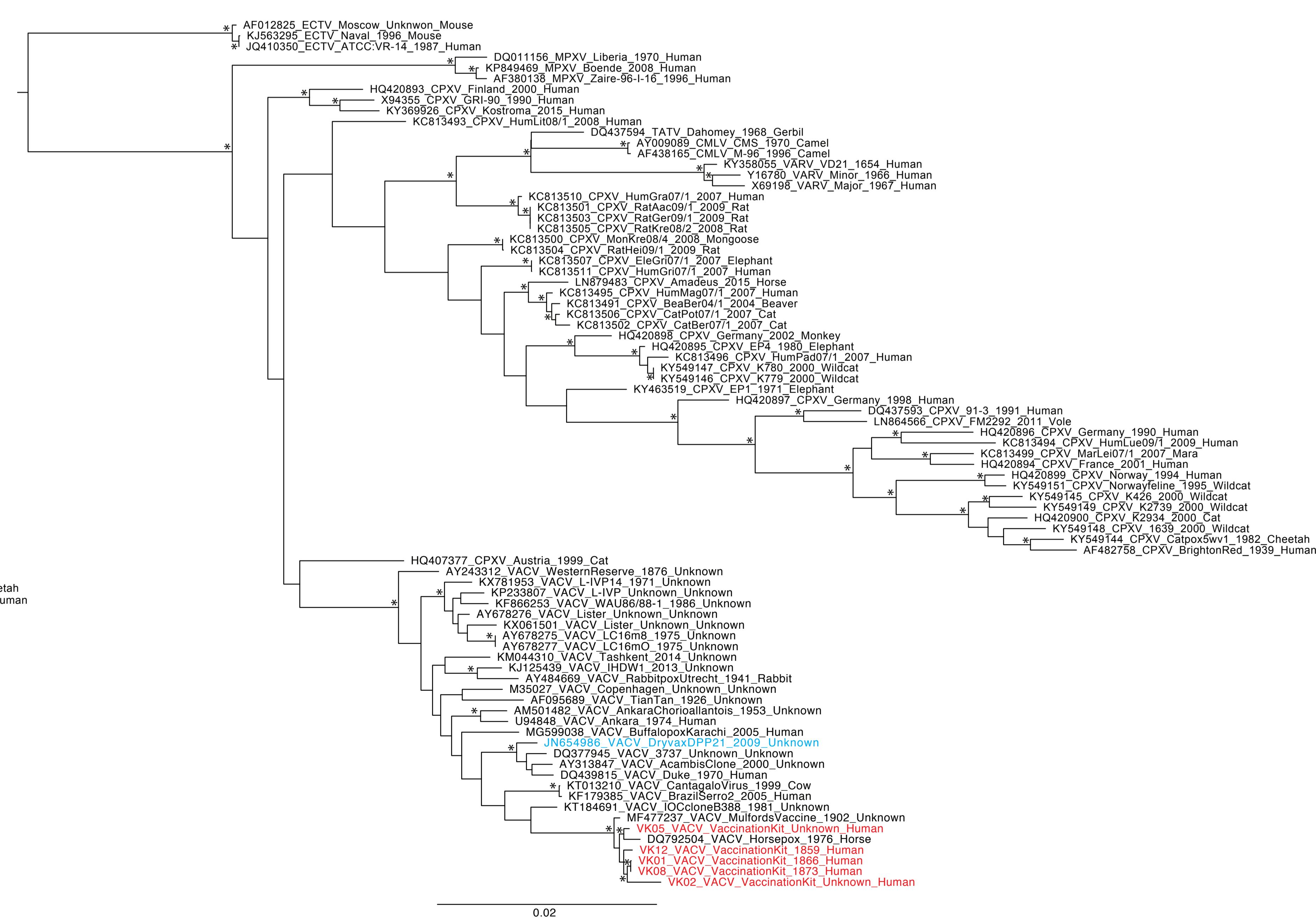

Supplement: Supplementary file 6 — Additional file 6: Figure S2. Maximum likelihood trees for Mutter vaccine strains called in reference to alternative VACV genomes. [file 13059_2020_2079_MOESM6_ESM.pdf]

A

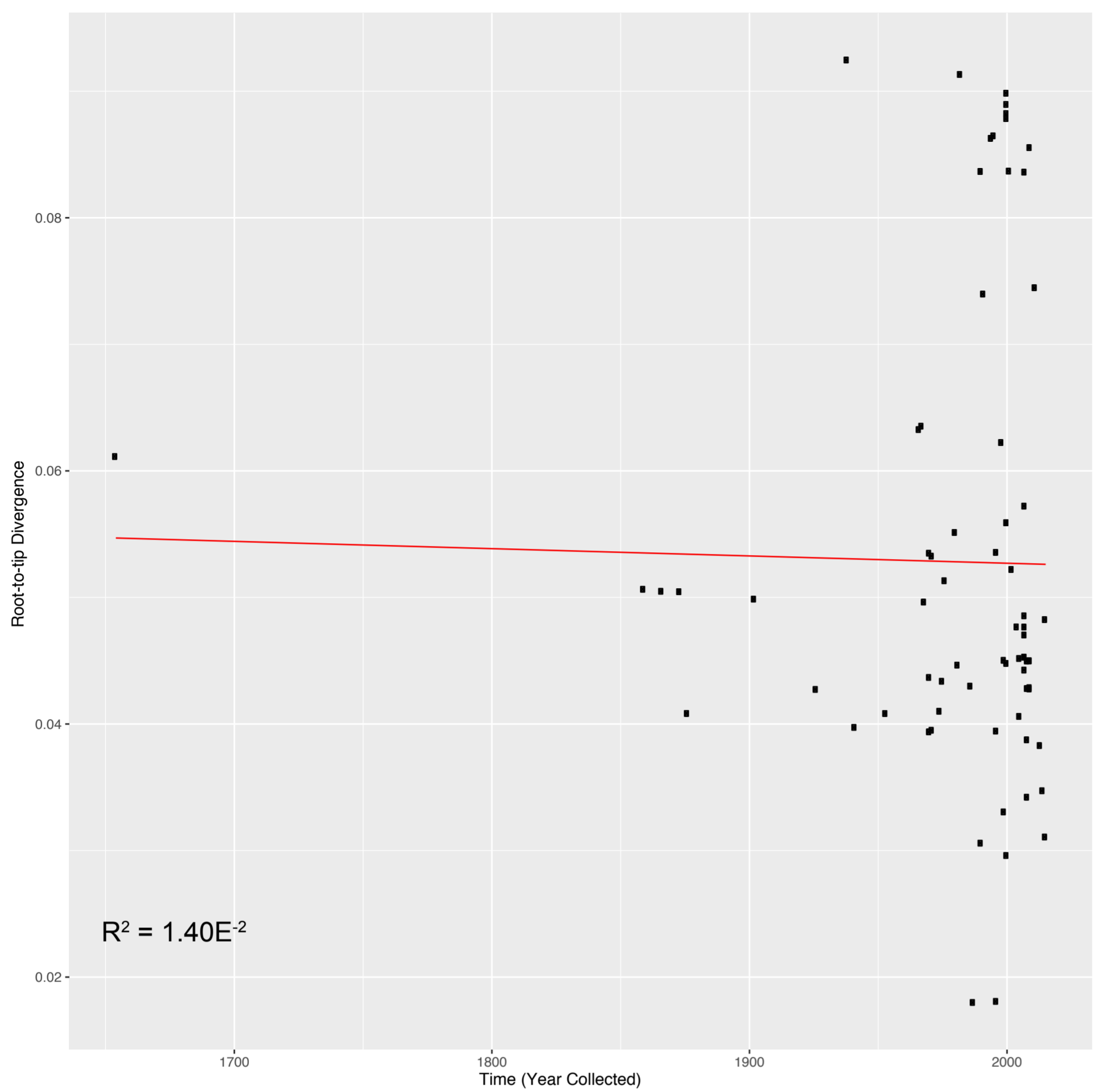

B

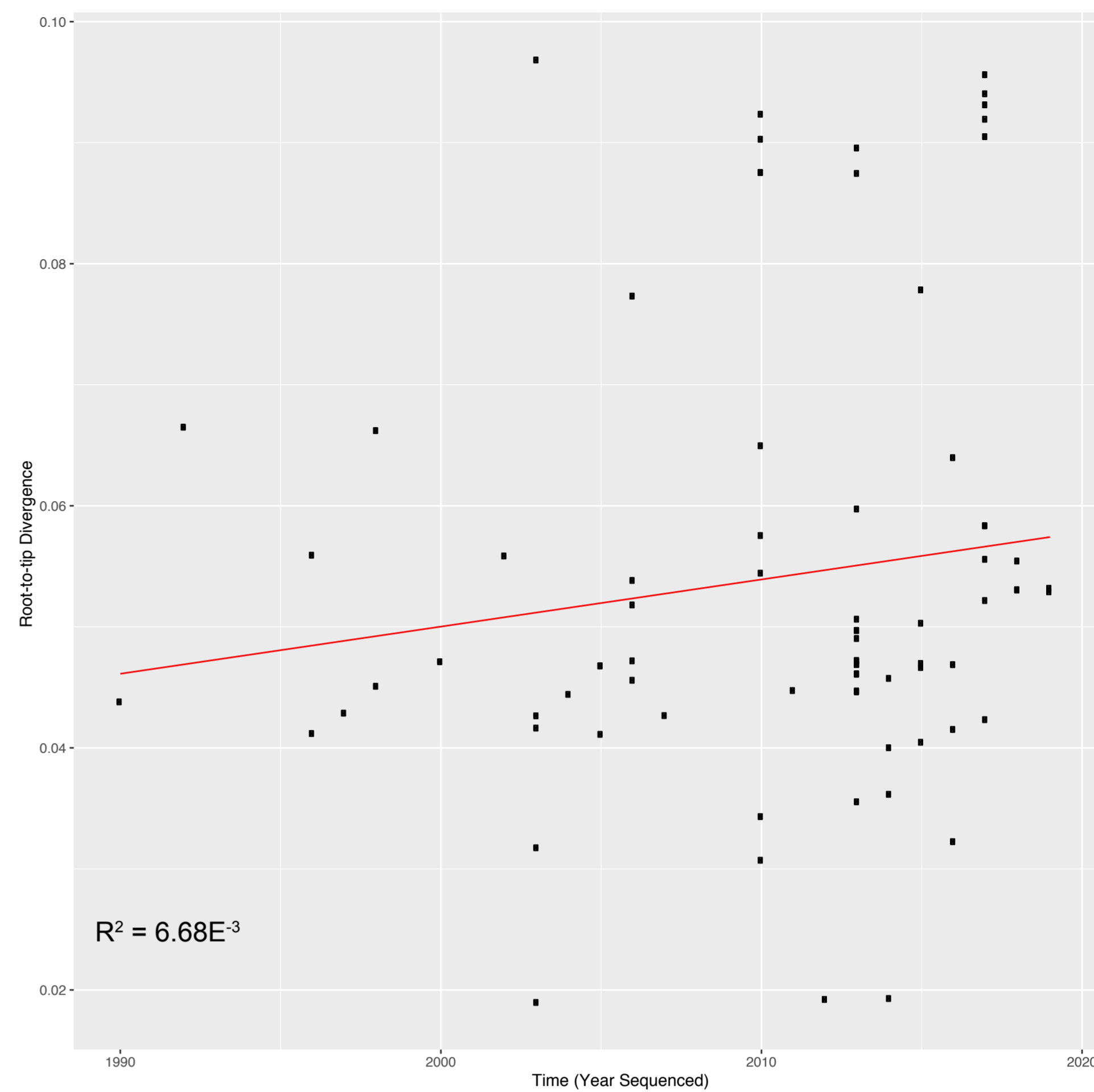

C

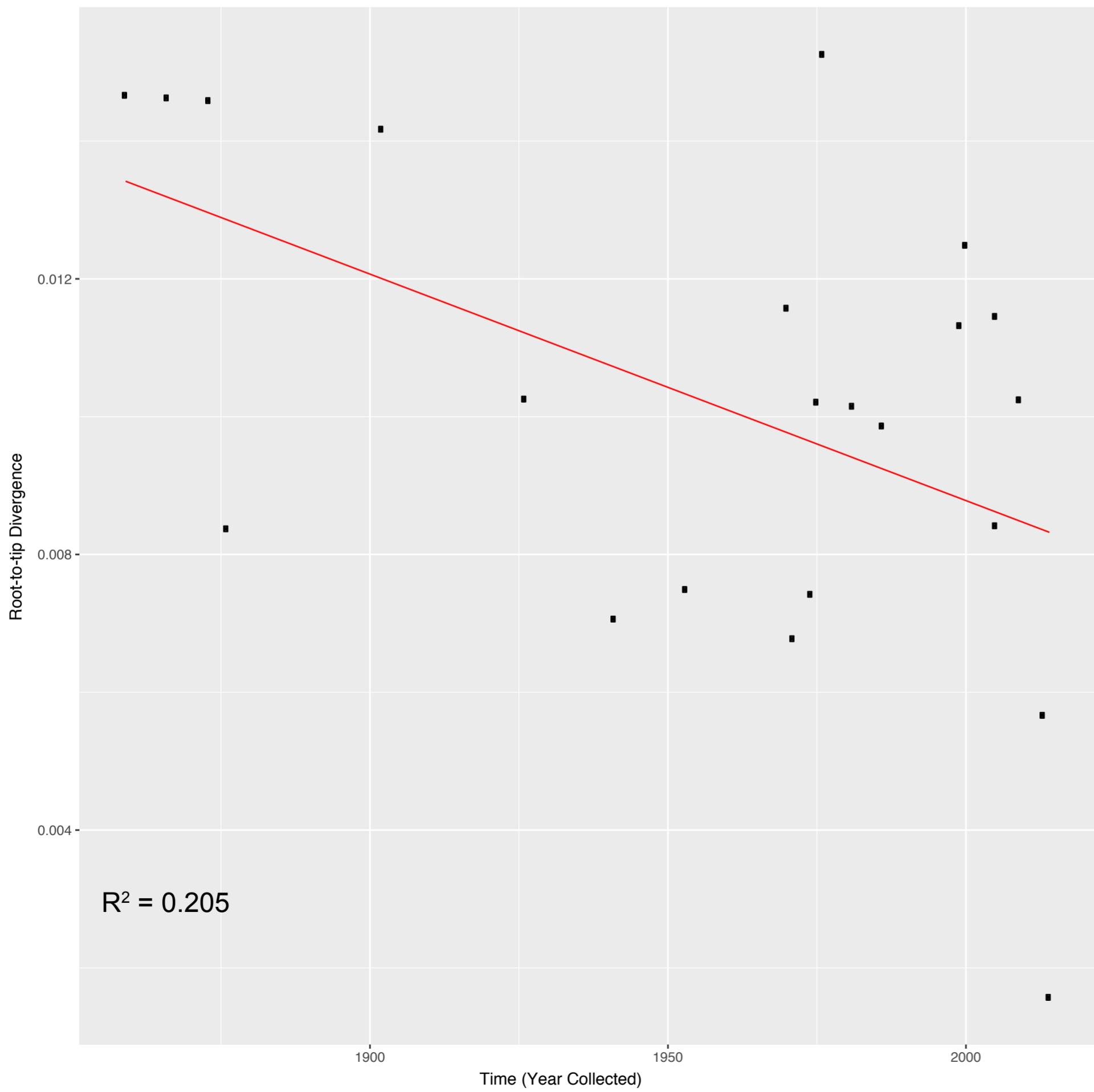

D

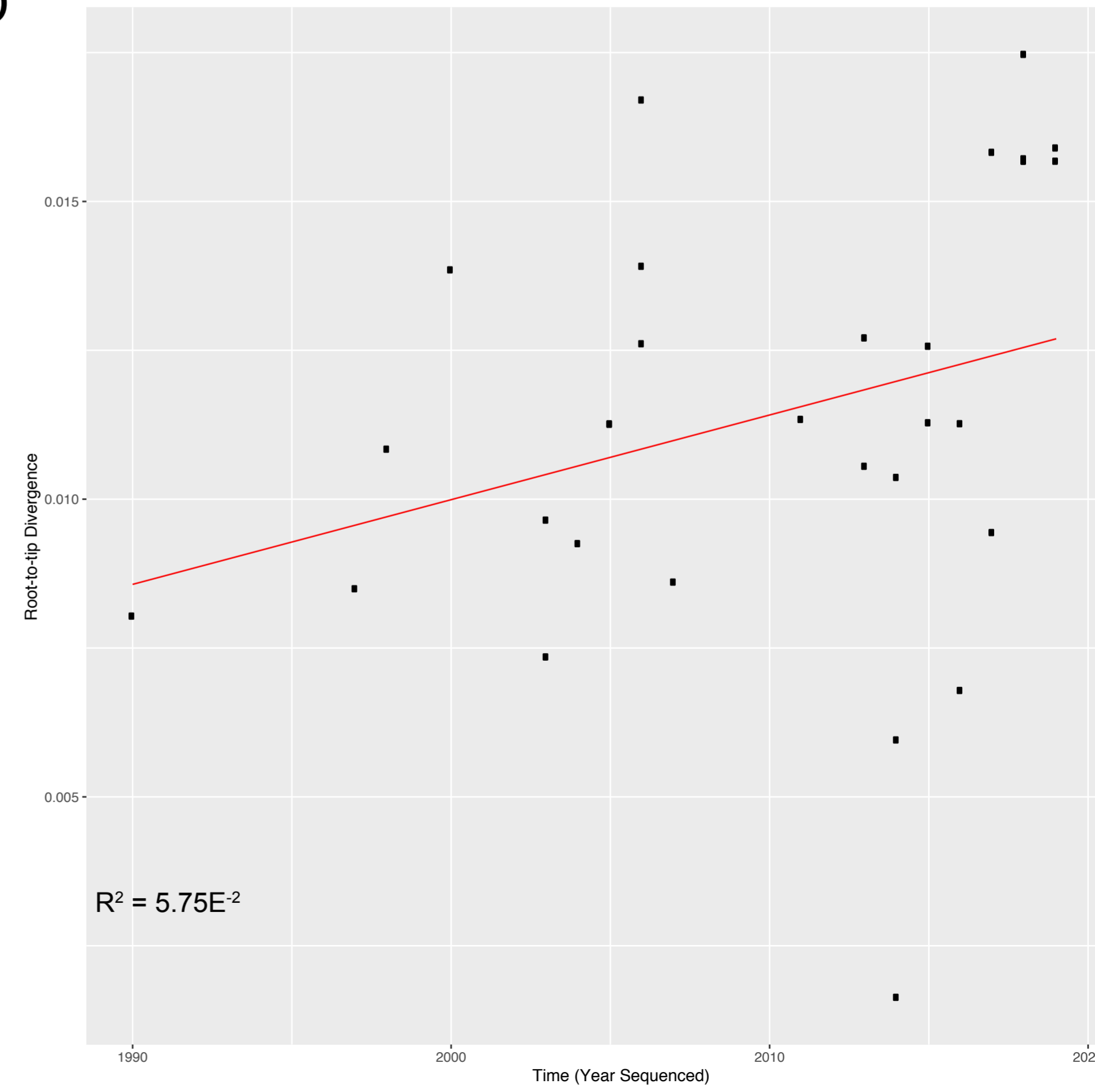

Supplement: Supplementary file 8 — Additional file 8: Figure S4. Regression analyses of root-to-tip genetic distance on the ML phylogeny against either year of collection or year of sequencing. [file 13059_2020_2079_MOESM8_ESM.pdf]

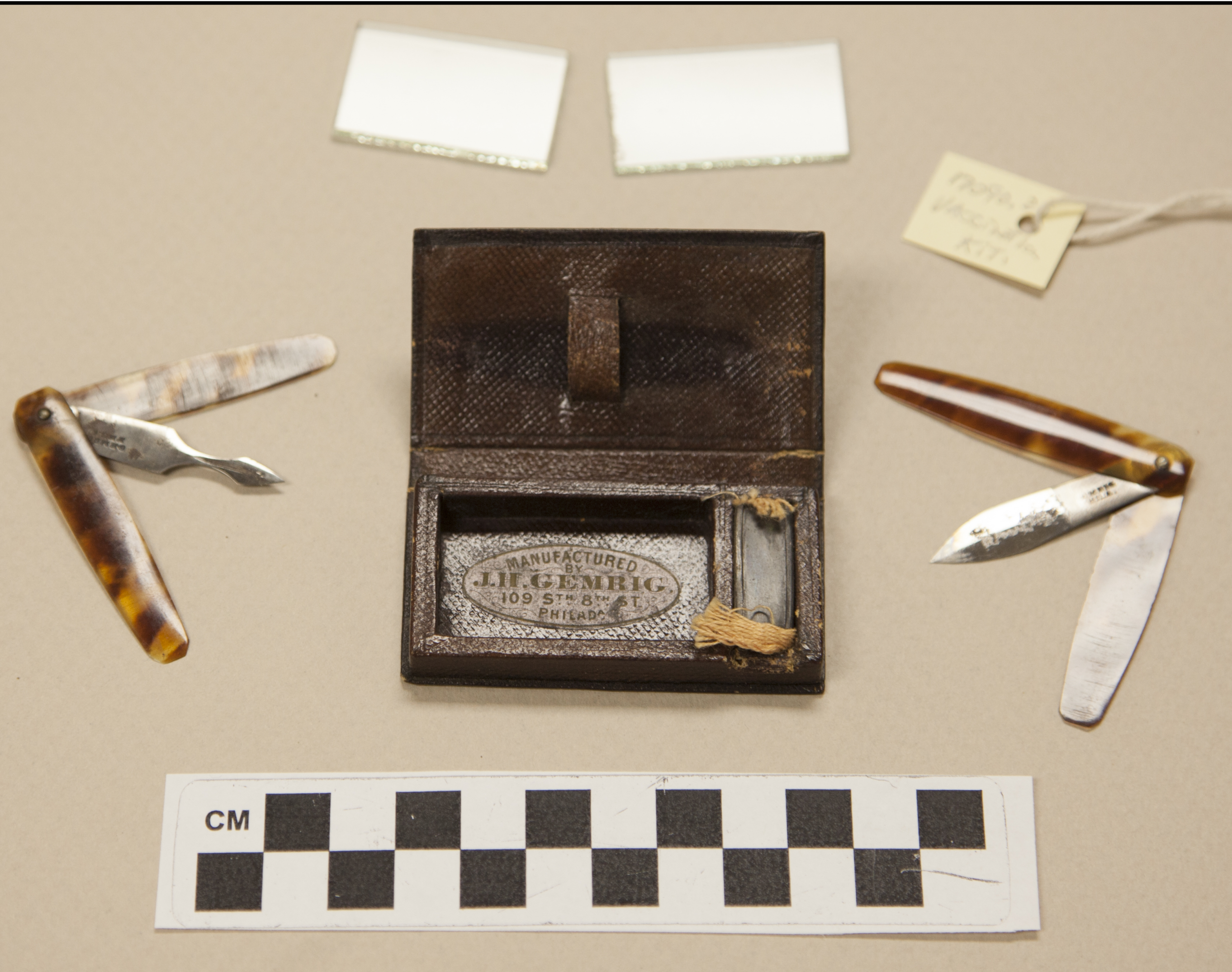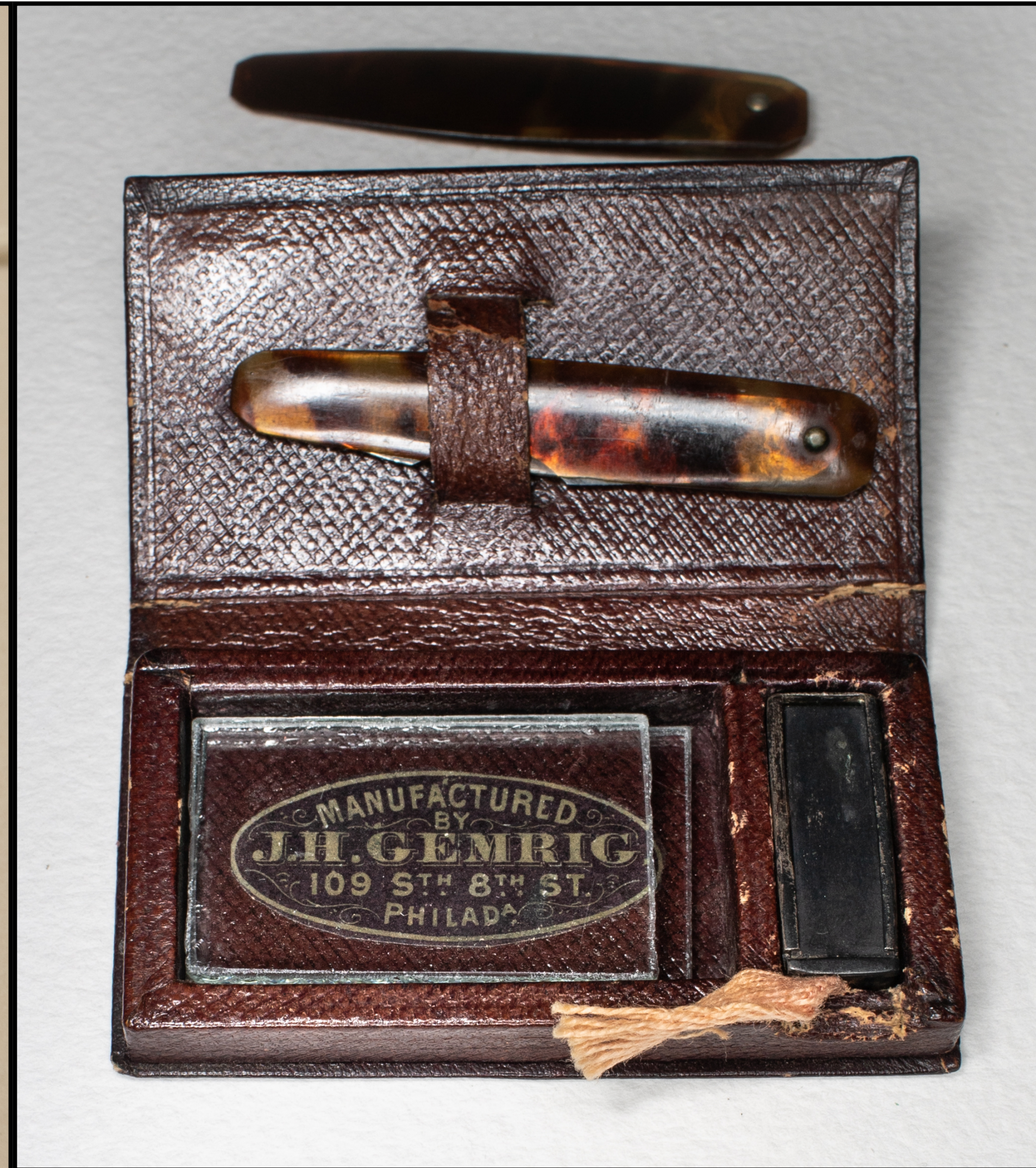

Supplement: Supplementary file 11 — Additional file 11: Figure S6. Mütter catalogue # 17090.29 before and after non-destructive sampling. [file 13059_2020_2079_MOESM11_ESM.pdf]

A

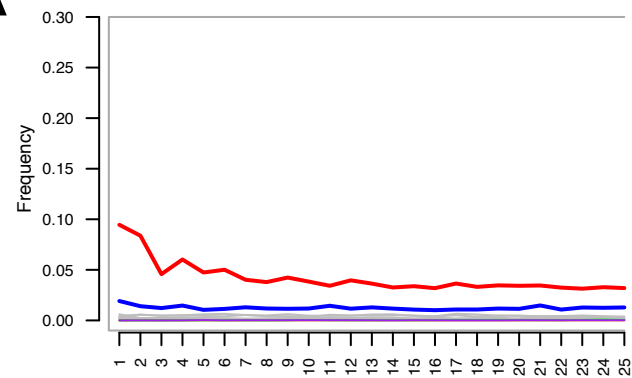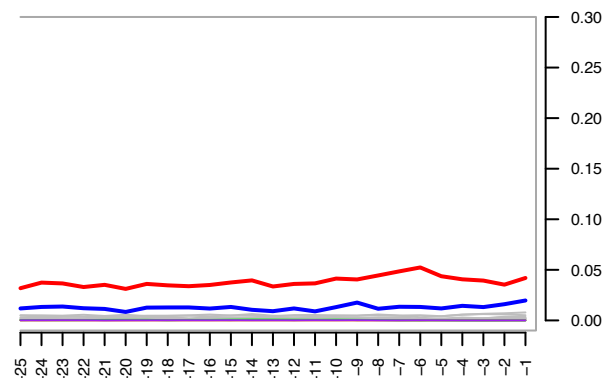

B

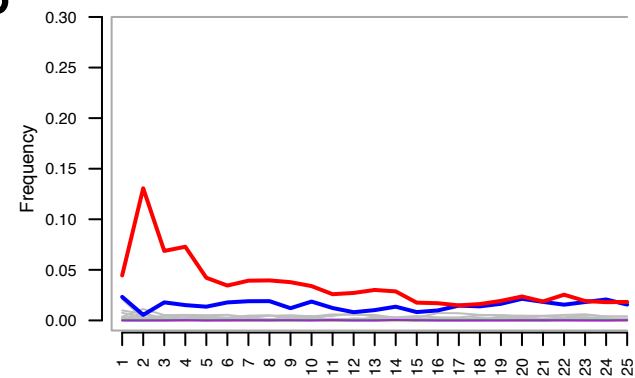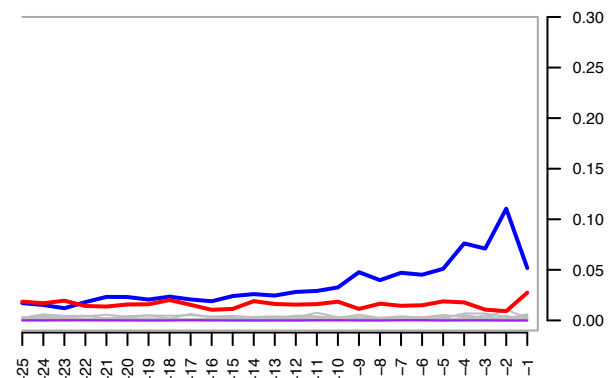

C

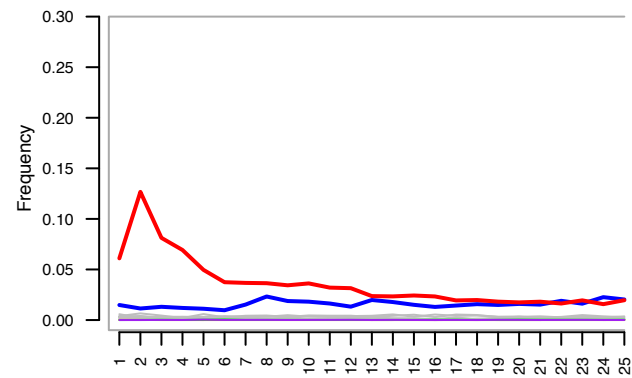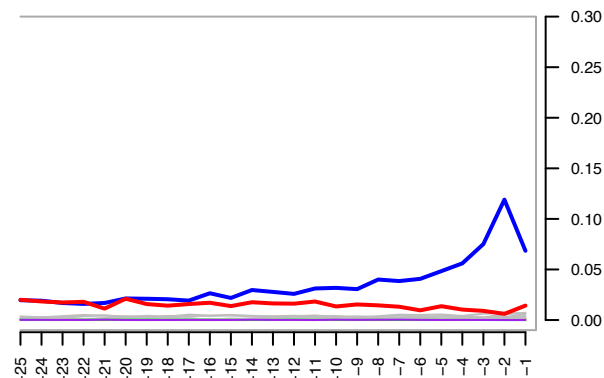

D

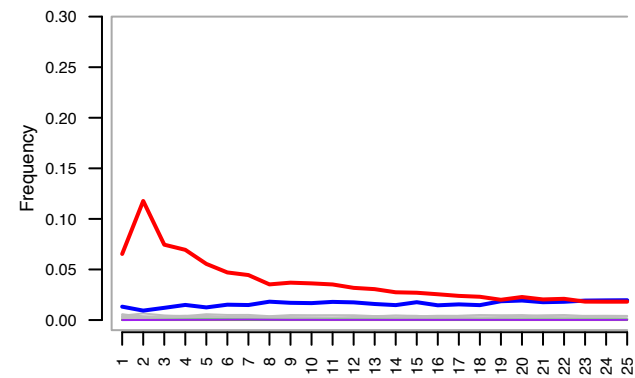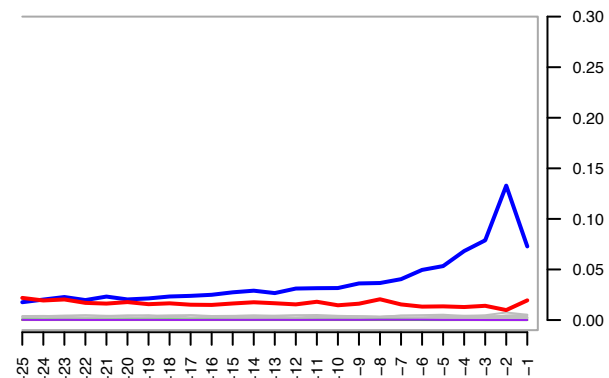

Supplement: Supplementary file 13 — Additional file 13: Figure S7. Comparison of terminal damage patterns of VK01. [file 13059_2020_2079_MOESM13_ESM.pdf]

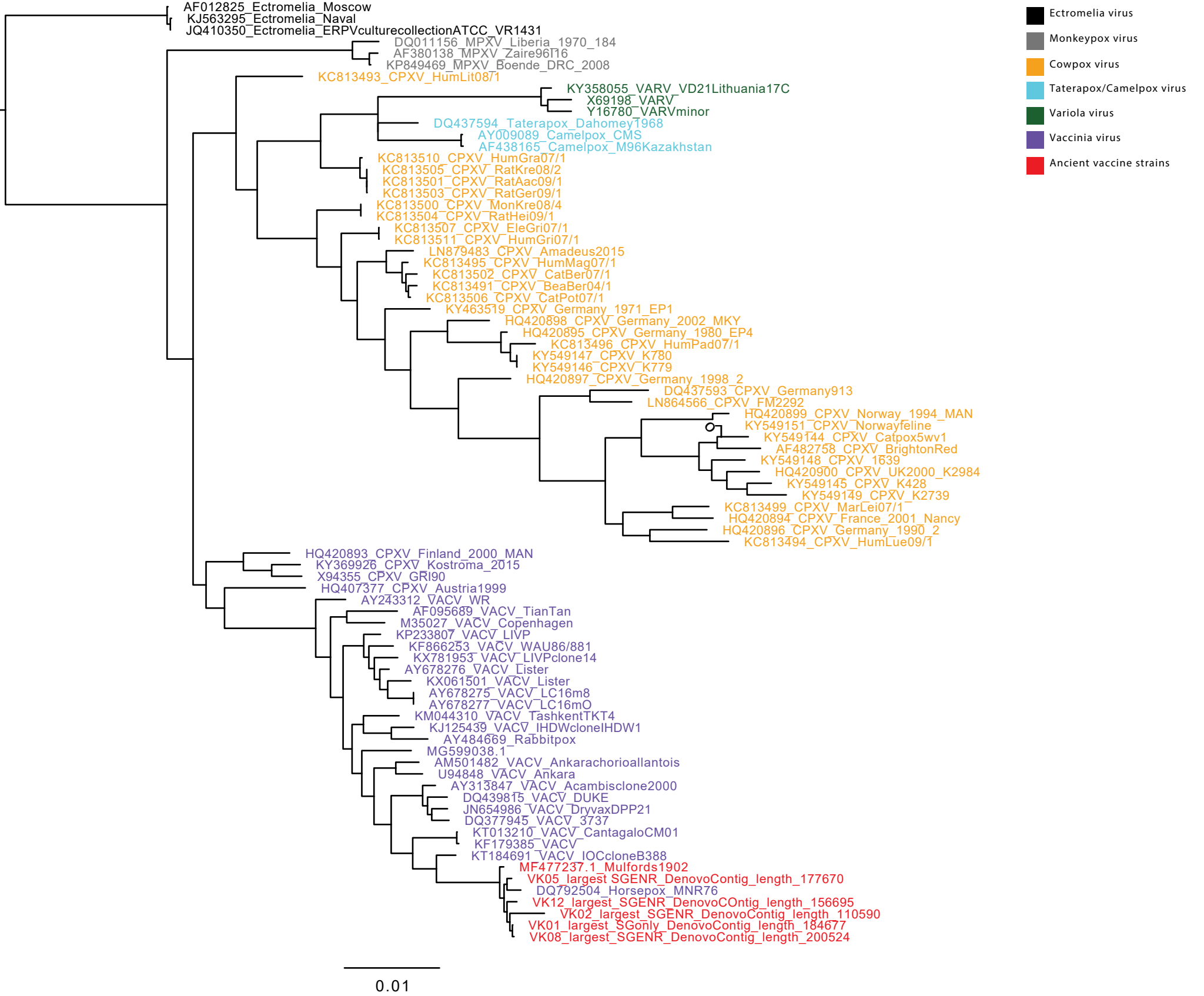

Supplement: Supplementary file 16 — Additional file 16: Figure S11. Maximum likelihood phylogenetic analysis of historical vaccine strains in relation to other OPXV using largest de novo assembled contig for each sample. [file 13059_2020_2079_MOESM16_ESM.pdf]
