# Supplementary material for: The origins and genomic diversity of American Civil War Era smallpox vaccine strains
Source: Genome Biol. 2020 Jul 20;21:175. doi: 10.1186/s13059-020-02079-z (PMC7370420; doi:10.1186/s13059-020-02079-z)
Supplement: Supplementary file 7 — Additional file 7: Figure S3. GrapeTree analysis of 79 OPXV. [file 13059_2020_2079_MOESM7_ESM.pdf]

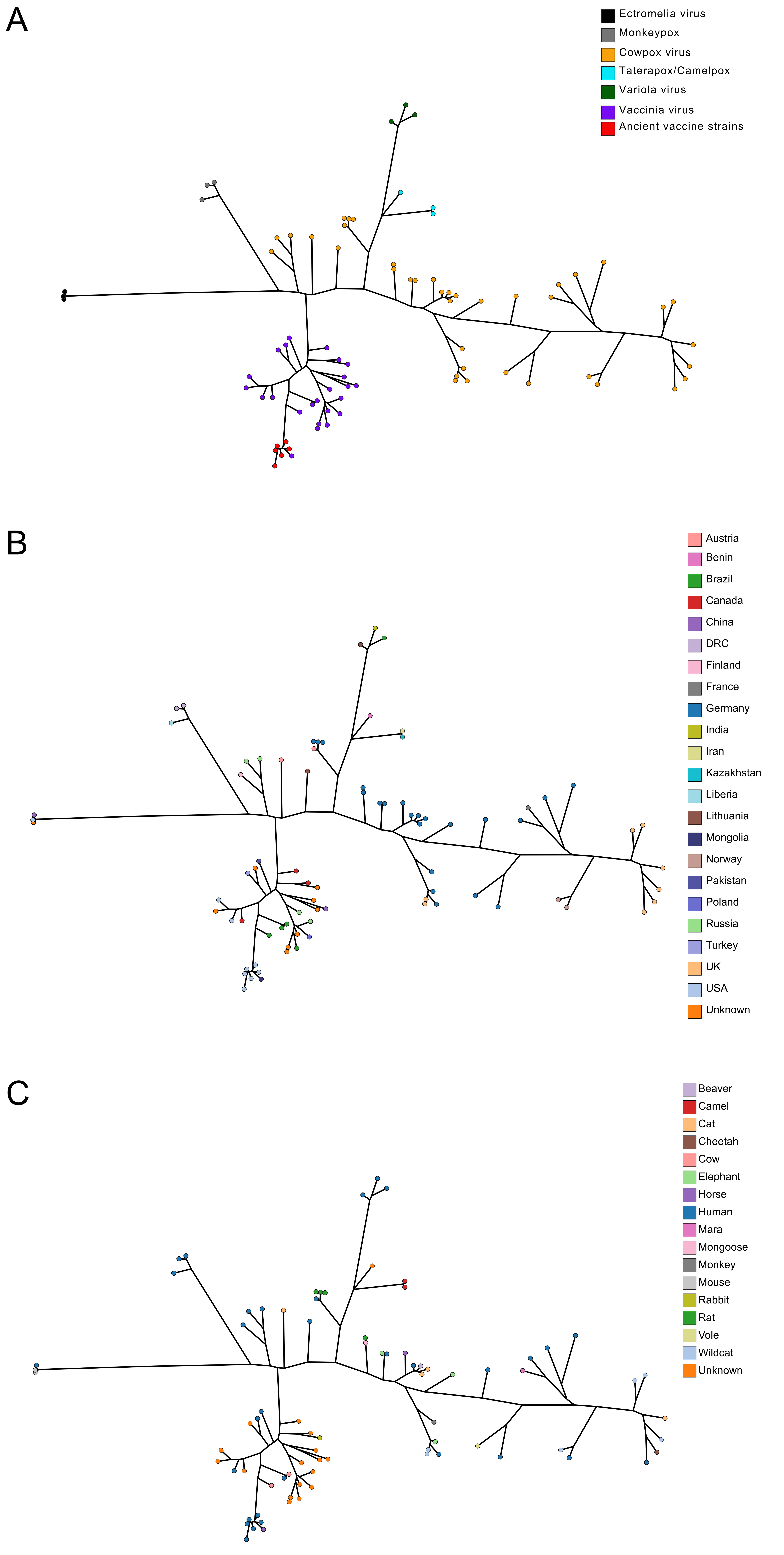
A
Ectromelia virus
Monkeypox
Cowpox virus
Taterapox/Camelpox
Variola virus
Vaccinia virus
Ancient vaccine strains
B
Austria
Benin
Brazil
Canada
China
DRC
Finland
France
Germany
India
Iran
Kazakhstan
Liberia
Lithuania
Mongolia
Norway
Pakistan
Poland
Russia
Turkey
UK
USA
Unknown
C
Beaver
Camel
Cat
Cheetah
Cow
Elephant
Horse
Human
Mara
Mongoose
Monkey
Mouse
Rabbit
Rat
Vole
Wildcat
Unknown
