# Supplementary material for: The origins and genomic diversity of American Civil War Era smallpox vaccine strains
Source: Genome Biol. 2020 Jul 20;21:175. doi: 10.1186/s13059-020-02079-z (PMC7370420; doi:10.1186/s13059-020-02079-z)
Supplement: Supplementary file 10 — Additional file 10: Figure S5. Partitioned maximum likelihood analysis of OPXV. [file 13059_2020_2079_MOESM10_ESM.pdf]

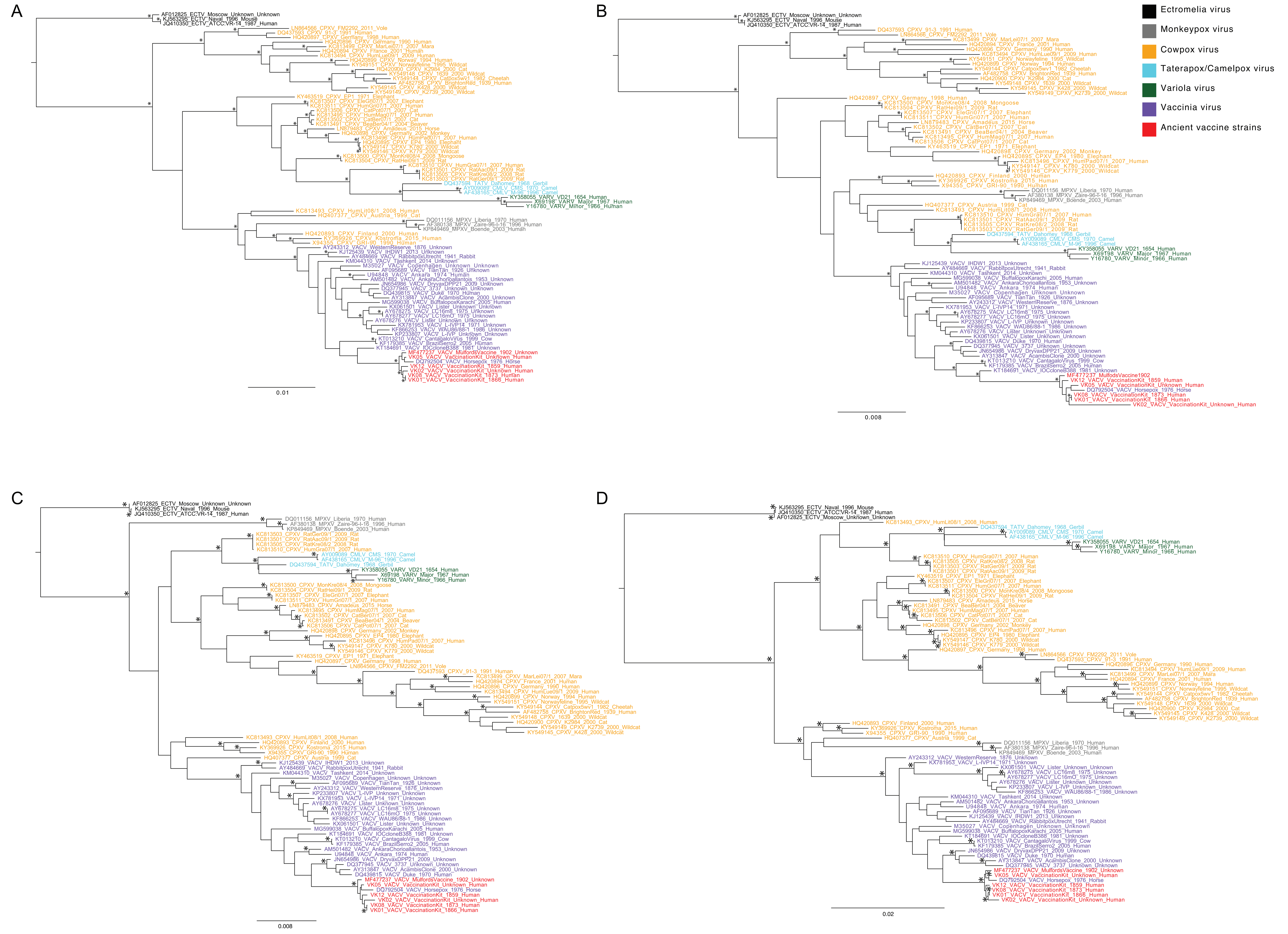

A
0.01
B
0.008
Ectromelia virus
Monkeypox virus
Cowpox virus
Taterapox/Camelpox virus
Variola virus
Vaccinia virus
Ancient vaccine strains
C
0.008
D
0.02
