# Supplementary material for: The origins and genomic diversity of American Civil War Era smallpox vaccine strains
Source: Genome Biol. 2020 Jul 20;21:175. doi: 10.1186/s13059-020-02079-z (PMC7370420; doi:10.1186/s13059-020-02079-z)
Supplement: Supplementary file 15 — Additional file 15: Figures S8–10. VK01 coverage relative to Copenhagen VACV strain M35027, GRI90 CPXV strain X93455, and Horsepox strain DQ792504. [file 13059_2020_2079_MOESM15_ESM.pdf]

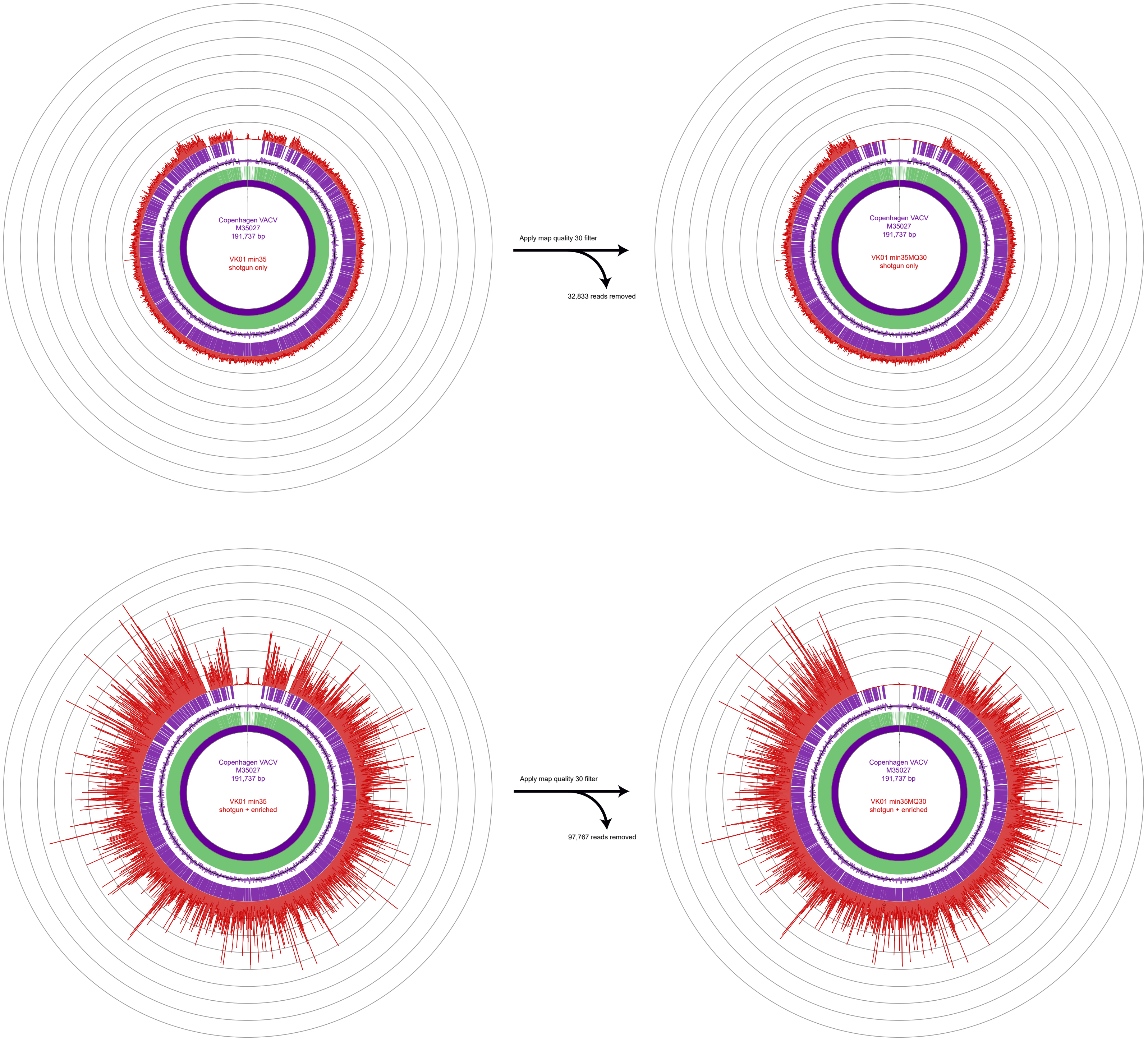
Copenhagen VACV
M35027
191,737 bp
VK01 min35
shotgun only
Apply map quality 30 filter
32,833 reads removed
Copenhagen VACV
M35027
191,737 bp
VK01 min35MQ30
shotgun only
Copenhagen VACV
M35027
191,737 bp
VK01 min35
shotgun + enriched
Apply map quality 30 filter
97,767 reads removed
Copenhagen VACV
M35027
191,737 bp
VK01 min35MQ30
shotgun + enriched

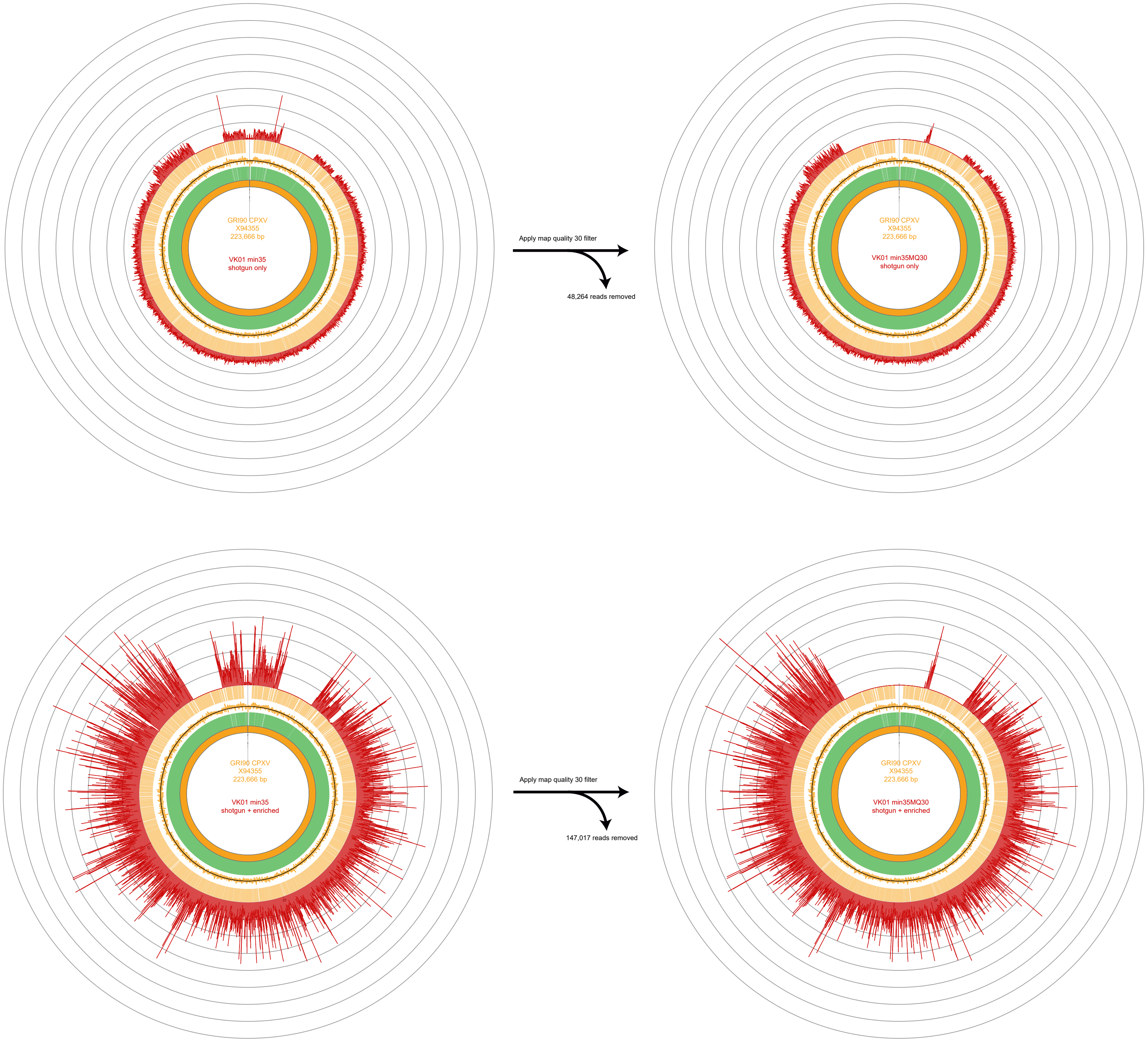

GRI90 CPXV
X94355
223,666 bp
VK01 min35
shotgun only
Apply map quality 30 filter
48,264 reads removed
GRI90 CPXV
X94355
223,666 bp
VK01 min35MQ30
shotgun only
GRI90 CPXV
X94355
223,666 bp
VK01 min35
shotgun + enriched
Apply map quality 30 filter
147,017 reads removed
GRI90 CPXV
X94355
223,666 bp
VK01 min35MQ30
shotgun + enriched

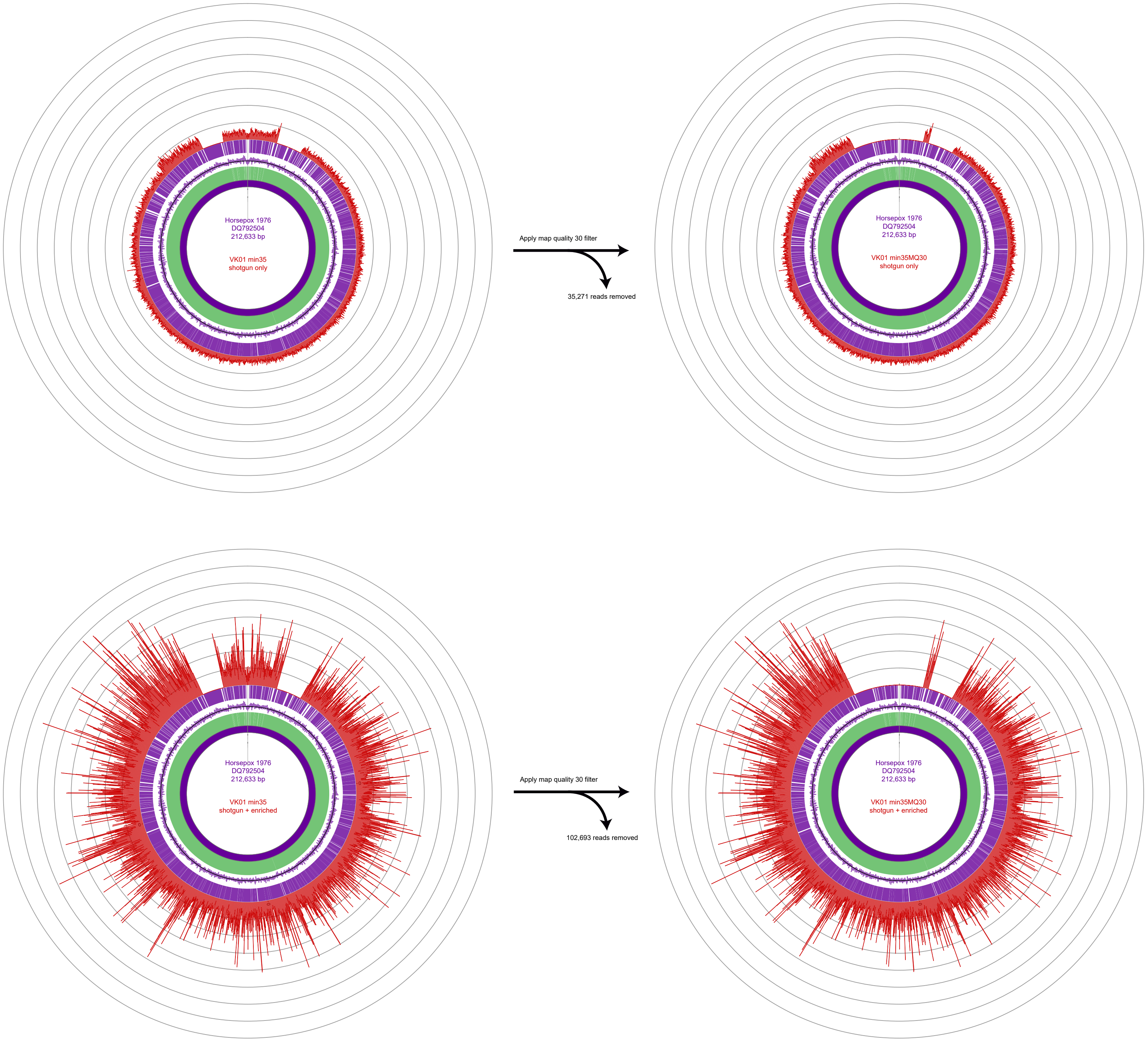
Horsepox 1976
DQ792504
212,633 bp
VK01 min35
shotgun only
Apply map quality 30 filter
35,271 reads removed
Horsepox 1976
DQ792504
212,633 bp
VK01 min35MQ30
shotgun only
Horsepox 1976
DQ792504
212,633 bp
VK01 min35
shotgun + enriched
Apply map quality 30 filter
102,693 reads removed
Horsepox 1976
DQ792504
212,633 bp
VK01 min35MQ30
shotgun + enriched
