# Supplementary material for: The origins and genomic diversity of American Civil War Era smallpox vaccine strains
Source: Genome Biol. 2020 Jul 20;21:175. doi: 10.1186/s13059-020-02079-z (PMC7370420; doi:10.1186/s13059-020-02079-z)
Supplement: Supplementary file 17 — Additional file 17. Review history. [file 13059_2020_2079_MOESM17_ESM.docx]

**Review history**

**1^st^ Round**

**Reviewer #1**

I found this a fascinating work that caught my attention by just reading the first two sentences of the Abstract. I think it is an original application of the second generation sequencing techniques, as well as metagenomic computational analysis, to an historical-medical problem -that of the variola virus strains used in vaccination in the mid 19th century- that otherwise could not be explored.

By sequencing several implements from vaccination kits used during or around the America Civil War, they were able to assemble de novo a virus genome (around 95%) from one of the samples at >10x coverage and map the others to this one with at least 10x coverage. They have also obtained, as expected, human data and used it for mtDNA haplotype determination (they are of European origin) and sex identification. With the genetic virus information they build up a phylogeny with modern strains that showed a cluster of all old strains with a commercially produced vaccination strain from Philadelphia in 1902 (plus, intriguingly, a modern strain isolated from a horse in Mongolia).

I think the study opens the possibility of studying similar tools used for vaccination in other countries and even continents that might be preserved in museums (the approach they did was non-destructive, which is of course another point of interest). This will likely help to expand our knowledge of the variola strains circulating prior to the eradication of the disease and also place value in some antique medical collections as reservoirs of future genetic research.

I don't have major concerns with this work, just few comments that are partly curiosity.

-In the Inorganic material-glass section, the authors used the Glock and Meyer method "B" but state they included also 0.05% Tween-20, which I think is not used in Glock and Meyer protocol; maybe they should say "modified from".

-In this section maybe the authors would like to cite Gelabert et al. 2016 PNAS; as far as I know this is the only other work that extracted DNA from antique glass slides, also in a similar way (immersing the slides overnight in Falcon tubes with the extraction buffer).

-In the section of Ancient DNA authentication, I think a reference on the deamination pattern of the reads would be needed; considering they mention the low values expected for samples that are <200 years, maybe this could be Sawyer et al. 2012 PlosOne.

-Also in this section, the pattern seen in Figure S1 and S2 shows, in general, a decrease of the C to T and G to A ratios in the very last nucleotide. I wonder if the authors have an explanation for this pattern that in most aDNA studies does not show this final one-nucleotide decrease.

-Considering there is a small but significant damage ratio (in some cases reaching 0.10 in the previous to last nucleotide), it would be interesting to know if the authors considered the possibility of trimming the ends of the reads prior to mapping or assembling. With high coverages of 10x this might not be a problem but maybe should be explained in the procedures.

Minor points:

-Line 187, Fig 2 Legend: "stains" -> "strains"

**Reviewer #2**

This is a very interesting paper that sheds further light on the origins of vaccinia virus. It would be of considerable interest to many readers.

However, there is an important problem with the way these sequences were filtered and assembled that could compromise the interpretation of these data. As noted in lines 557-572, VACV strain Copenhagen (and VARV) were used to retrieve sequence reads out of the mix of mostly human DNA. However, given the interest in the relationship to cowpox viruses, this approach would likely eliminate any reads that mapped to those telomeric regions of Orthopoxviruses that differentiate cowpox strains from vaccinia virus strains. I've attached a whole genome alignment that illustrates the problem and shows the reads this approach could lose. The authors then go on to build phylogenies using the central and most highly conserved portion of Orthopoxvirus genomes. Unfortunately what they may have done is toss out important telomeric sequence information. This approach uses circular reasoning (VACV is used to retrieve sequences that not-surprisingly look like VACV), and the error is common to all of the genome assemblies.

One can illustrate how this leads to problems because the authors make the claim that Mongolian HSPV is a vaccinia virus (lines 178-9). I'd argue this doesn't reflect the evidence from genomics. All one has to do is look at the whole-genome alignments (see attached figure) and see that HSPV encodes extra sequences that are characteristic of many different vaccinia-like cowpox strains, and which have been lost from all known modern VACV strains. I don't dispute that the central portion of HSPV shows that it is a cowpox strain most closely related to VACV, but the ends show it's not an escaped modern VACV. Indeed there's nothing uniquely special about the Mongolian HSPV strain. If it had not been sequenced any one of many other VACV-like cowpox strains would show up in these analyses as resembling VACV.

If a cowpox genome was used to filter the sequence reads, would one capture these extra sequences? Do the assemblies terminate at sites where VACV diverges from cowpox strains? This should be re-tested.

There is one other matter that deserves some further comment from the authors. It relates to the placement of these sequences in the phylograms. Branch lengths are difficult to interpret where viruses are concerned and the authors are commended for having taken a rigorous approach to assembling these trees. Nevertheless it is VERY difficult to see how a horsepox virus, separated by ~100 years in time and isolated halfway around the world, could show such a close resemblance to these new strains. By comparison there is more sequence divergence detected among the quasispecies that can be isolated from a 20th century stock of Dryvax (DPP21, Duke, AK2K, and 3737). Something somewhere just doesn't add up and it casts doubt on these methods. Comments?

[I suppose it is conceivable that the Mongolian HSPV is a VACV-cowpox recombinant that's retained the cowpox telomeres. However it is still difficult to picture how the sequence in the central VACV-like middle could have suffered so little sequence drift unless Mongolia was (until late in the eradication campaign) using an old French vaccine strain. Another possibility is that the "HSPV" specimen has been mixed up with a very old still cowpox-like archival vaccine, perhaps at the time of isolation.]

On a minor note it's not clear that Figure S4 shows anything useful, nor would one expect to see any such correlations. A figure showing a representative example of a typical region of sequence alignment, and the read quality would be more informative.

**Reviewer #3**

The manuscript describes the detection and reconstruction of poxvirus genomes from historic samples taken form so-called „vaccination kits" from the 19th century in the US.

Ancient DNA analysis is especially suitable for the reconstruction of DNA viruses and allows new insights into the evolution of these viruses.

The study is straight forward, interesting and important and provides additional data, which are for all reconstructed sequences in line with the data from related vaccine strains from the beginning of the 20th century. A main new conclusion is the obvious „common wisdom" and also a common source of material which was used to vaccinate people against smallpox even more than 150 years ago.

Overall, there are several points which should be taken into consideration:

1. Line 42-51: The Abstract should provide more data and information about the results. How many genomes could be reconstructed? What are the main (new) conclusions?

2. Line 55: The genus orthopoxvirus should be written in italics since it is used here together with the term „genus"

3. Line 57: it should read „human infectious disease"

4. Background in general: The modified vaccinia virus Ankara should be mentioned as one of the strains which is now fully characterized as one of the more modern poxvirus vaccine strains.

5. Line 75: It should be mentioned that nomenclature of CPXV and related viruses is inconclusive and needs novel concepts.

6. Line 104: why is there so much human DNA detected in the samples from the „vaccination kits"? Please explain in more detail.

7. Line 104: Why is the small number of bacterial reads unexpected? Please explain

8. Line 106: Please mention the enrichment method here.

9. 154: I would suggest to assemble also the enriched reads. For a draft analysis the quality should be sufficient and could provide further insights.

10. Figure 2a: The newly described CPXV from voles have to be also included (e.g. also the Ectromelia like CPXV from bank voles)

11. Line 598: The metagenome analysis should be repeated and improved by using a modern metagenome workflow. This should be done to be sure that there is no further hits for viruses or bacteria.

**Response to reviewers**

Reviewer #1: I found this a fascinating work that caught my attention by just reading the first two sentences of the Abstract. I think it is an original application of the second generation sequencing techniques, as well as metagenomic computational analysis, to an historical-medical problem -that of the variola virus strains used in vaccination in the mid 19th century- that otherwise could not be explored.

By sequencing several implements from vaccination kits used during or around the America Civil War, they were able to assemble de novo a virus genome (around 95%) from one of the samples at >10x coverage and map the others to this one with at least 10x coverage. They have also obtained, as expected, human data and used it for mtDNA haplotype determination (they are of European origin) and sex identification.

With the genetic virus information they build up a phylogeny with modern strains that showed a cluster of all old strains with a commercially produced vaccination strain from Philadelphia in 1902 (plus, intriguingly, a modern strain isolated from a horse in Mongolia).

I think the study opens the possibility of studying similar tools used for vaccination in other countries and even continents that might be preserved in museums (the approach they did was non-destructive, which is of course another point of interest). This will likely help to expand our knowledge of the variola strains circulating prior to the eradication of the disease and also place value in some antique medical collections as reservoirs of future genetic research.

Response: We thank the reviewer for these positive comments.

I don't have major concerns with this work, just few comments that are partly curiosity.

-In the Inorganic material-glass section, the authors used the Glock and Meyer method "B" but state they included also 0.05% Tween-20, which I think is not used in Glock and Meyer protocol; maybe they should say "modified from".

Response: This has been added as suggested and clarified in the methods section.

-In this section maybe the authors would like to cite Gelabert et al. 2016 PNAS; as far as I know this is the only other work that extracted DNA from antique glass slides, also in a similar way (immersing the slides overnight in Falcon tubes with the extraction buffer).

Response: This has been added as suggested.

-In the section of Ancient DNA authentication, I think a reference on the deamination pattern of the reads would be needed; considering they mention the low values expected for samples that are <200 years, maybe this could be Sawyer et al. 2012 PlosOne.

Response: This has been added as suggested

-Also in this section, the pattern seen in Figure S1 and S2 shows, in general, a decrease of the C to T and G to A ratios in the very last nucleotide. I wonder if the authors have an explanation for this pattern that in most aDNA studies does not show this final one-nucleotide decrease.

Response: Despite our best efforts (as detailed in Figure S2), we do not have a satisfactory explanation for the decrease in the C to T and G to A ratio at the terminal nucleotide. Our suspicion is that, given the richness of molecules available within this library, the enzymes are selectively ligating adapters to molecules without damage in the most terminal position. However, our experimentations in increasing the adapter concentration relative to library input were inconclusive (Figure S2 C&D).

-Considering there is a small but significant damage ratio (in some cases reaching 0.10 in the previous to last nucleotide), it would be interesting to know if the authors considered the possibility of trimming the ends of the reads prior to mapping or assembling. With high coverages of 10x this might not be a problem but maybe should be explained in the procedures.

Response: We chose not to trim the ends of our reads prior to mapping. As the reviewer notes, we have very high coverage for all 5 novel genomes produced in this work (all >146x coverage) and we do not believe terminal damage will have affected any of our consensus base calls.

Minor points:

-Line 187, Fig 2 Legend: "stains" -> "strains"

Response: Corrected.

Reviewer #2:

This is a very interesting paper that sheds further light on the origins of vaccinia virus. It would be of considerable interest to many readers.

However, there is an important problem with the way these sequences were filtered and assembled that could compromise the interpretation of these data. As noted in lines 557-572, VACV strain Copenhagen (and VARV) were used to retrieve sequence reads out of the mix of mostly human DNA. However, given the interest in the relationship to cowpox viruses, this approach would likely eliminate any reads that mapped to those telomeric regions of Orthopoxviruses that differentiate cowpox strains from vaccinia virus strains. I've attached a whole genome alignment that illustrates the problem and shows the reads this approach could lose. The authors then go on to build phylogenies using the central and most highly conserved portion of Orthopoxvirus genomes. Unfortunately what they may have done is toss out important telomeric sequence information. This approach uses circular reasoning (VACV is used to retrieve sequences that not-surprisingly look like VACV), and the error is common to all of the genome assemblies.

Response: The reviewer has misunderstood aspects of our analysis. First, using the procedure described we were able to construct a full-length virus genome. Under the reviewer's logic this would not have happened as we would have failed to obtain the supposedly divergent telomeric regions that only map to CPXV. This didn't happen. Is the reviewer then suggesting that our sequences contain both VACV-like and CPXVlike reads? We don't see how this is possible. As an aside, the reference to cowpox is misleading as CPXV is not a monophyletic group and so different assemblies would be expected depending on which specific CPXV was used. Second, we performed a de novo assembly of sequence VK01 and then used this as a guide for the assembly of the other sequences. Third, we did not exclude the telomeric regions from the phylogenetic analysis. Rather, we performed a complete genome sequence alignment and then removed poorly aligned regions across the entire genome using GBlocks. This exercise is hugely important because the terminal regions are extremely difficult to align. Indeed, in many cases it is impossible to accurately assign positional homology. Evolutionary relationships can only safely be assigned when where is clear positional homology.

We have now performed a second, more relaxed, GBlocks analysis that allows gaps, resulting a new sequence alignment of 179,308 nt (as opposed to 134,607 nt used in the paper). As can be seen in the attached tree, the key evolutionary relationships are unchanged using this new alignment: the Civil War vaccine strains cluster closely with the horsepox virus and this group (shown in red) falls squarely within the VACV clade.

One can illustrate how this leads to problems because the authors make the claim that Mongolian HSPV is a vaccinia virus (lines 178-9). I'd argue this doesn't reflect the evidence from genomics. All one has to do is look at the whole-genome alignments (see attached figure) and see that HSPV encodes extra sequences that are characteristic of many different vaccinia-like cowpox strains, and which have been lost from all known modern VACV strains. I don't dispute that the central portion of HSPV shows that it is a cowpox strain most closely related to VACV, but the ends show it's not an escaped modern VACV. Indeed there's nothing uniquely special about the Mongolian HSPV strain. If it had not been sequenced any one of many other VACV-like cowpox strains would show up in these analyses as resembling VACV.

Response: Please see our response above. In addition, visual inspection shows that positional homology is extremely difficult to determine in the terminal regions so what we distrust any evolutionary inferences made on these data. Our visual inspection also reveals indels that clearly link HSPV to VACV in contradiction of what the reviewer says.

More broadly, our paper is about the 19th century vaccine strains, not horsepox (which we obviously did not sequence). Whatever the reviewer may feel, every previous evolutionary analysis of orthopoxvirus evolution has clearly placed HSPV within the VACV group, including the original HPSV genomic study of Tulman et al. (J.Virol. 80, 9244-9258), and HSPV is routinely described as a variant of VACV.

If a cowpox genome was used to filter the sequence reads, would one capture these extra sequences? Do the assemblies terminate at sites where VACV diverges from cowpox strains? This should be re-tested.

Response: Again, please see our response above and recall that CPXV strains are highly diverse and do not form a monophyletic group, with some strains more closely related to VACV than others. Hence, which CPXV strain is chosen will have a major impact on the assemblies.

There is one other matter that deserves some further comment from the authors. It relates to the placement of these sequences in the phylograms. Branch lengths are difficult to interpret where viruses are concerned and the authors are commended for having taken a rigorous approach to assembling these trees. Nevertheless it is VERY difficult to see how a horsepox virus, separated by ~100 years in time and isolated halfway around the world, could show such a close resemblance to these new strains. By comparison there is more sequence divergence detected among the quasispecies that can be isolated from a 20th century stock of Dryvax (DPP21, Duke, AK2K, and 3737). Something somewhere just doesn't add up and it casts doubt on these methods. Comments?

Response: Although these sequences look similar this is in fact deceptive as poxviruses have large genomes and evolve relatively slowly. In reality, the genetic distance between these vaccine strains and horsepox virus is very close to that predicted given their time of separation. This is easy to show with some simple calculations. Our sequence alignment (following GBlocks pruning) is 134,607 nt. The horsepox virus and its most closely related 19th century vaccine strain differ by 238 nucleotide changes, giving a genetic distance of 0.00177. Although we don't know when these viruses diverged, the highest rate estimate (which is the most conservative option given the idea proposed by the reviewer) is obtained when the vaccine strain (~1860) is directly ancestral to the horsepox strain (1976): this gives a divergence time of 116 years. Under these assumptions the evolutionary rate would be 0.00177/116 = 1.5 x 10-5 nucleotide substitutions per site, per year. This compares very favourably to previous estimates of evolutionary rates in poxviruses, including VARV (Duggan et al. Curr.Biol. 26, 3407-3412, 2016; Kerr et al. PLoS Pathog. 8:e1002950, 2012). A more realistic relationship between the Civil War vaccine strains and HSPV - in which they share a common ancestor rather than being direct ancestor-descendant - would produce a lower evolutionary rate that is even closer to those observed in other orthopoxviruses.

Finally, and crucially, the reviewer forgets that another group independently (i.e. in a different laboratory) obtained an orthopoxvirus from an early 20th century vaccination kit (Schrick et al. NEJM 377, 1491-1492, 2017). As this falls in the same cluster as our virus it acts an essential verification of our work: both the close relationship to HSPV and the observed branch lengths. This has been noted in the revised version of the paper.

[I suppose it is conceivable that the Mongolian HSPV is a VACV-cowpox recombinant that's retained the cowpox telomeres. However it is still difficult to picture how the sequence in the central VACV-like middle could have suffered so little sequence drift unless Mongolia was (until late in the eradication campaign) using an old French vaccine strain. Another possibility is that the "HSPV" specimen has been mixed up with a very old still cowpox-like archival vaccine, perhaps at the time of isolation.]

Response: Please see our responses above. No one has ever doubted the close relationship between HSPV and VACV, and our paper is not about HSPV.

On a minor note it's not clear that Figure S4 shows anything useful, nor would one expect to see any such correlations. A figure showing a representative example of a typical region of sequence alignment, and the read quality would be more informative.

Response: We have to disagree with the reviewer here. This figure very clearly shows the lack of temporal structure in these data meaning that we cannot perform a molecular clock dating analysis. Interested readers can easily download the sequence and repeat the alignment themselves.

Reviewer #3:

The manuscript describes the detection and reconstruction of poxvirus genomes from historic samples taken form so-called „vaccination kits" from the 19th century in the US.

Ancient DNA analysis is especially suitable for the reconstruction of DNA viruses and allows new insights into the evolution of these viruses.

The study is straight forward, interesting and important and provides additional data, which are for all reconstructed sequences in line with the data from related vaccine strains from the beginning of the 20th century. A main new conclusion is the obvious „common wisdom" and also a common source of material which was used to vaccinate people against smallpox even more than 150 years ago.

Overall, there are several points which should be taken into consideration:

1. Line 42-51: The Abstract should provide more data and information about the results. How many genomes could be reconstructed? What are the main (new) conclusions?

Response: Unfortunately, as our paper is submitted as a 'Short Report' to Genome Biology we are only allowed an Abstract of 100 words. Given these severe length constraints, and that we are at 100 words already, it is not clear what we can meaningfully add to the Abstract.

2. Line 55: The genus orthopoxvirus should be written in italics since it is used here together with the term „genus"

Response: Corrected.

3. Line 57: it should read „human infectious disease"

Response: Corrected.

4. Background in general: The modified vaccinia virus Ankara should be mentioned as one of the strains which is now fully characterized as one of the more modern poxvirus vaccine strains.

Response: As this information is not pertinent to the paper, and because of major length restrictions, we have not been able to mention this in the revised version of the paper.

5. Line 75: It should be mentioned that nomenclature of CPXV and related viruses is inconclusive and needs novel concepts.

Response: We agree, although as this is not the point of the point of the paper and because of length constraints we unfortunately do not have the space to cover this.

6. Line 104: why is there so much human DNA detected in the samples from the „vaccination kits"? Please explain in more detail.

Response: These vaccination kits do not represent a commercial or purified vaccine. They date from a period when vaccines were still propagated within human bodies so that the biological material found in these kits originated from human patients. The scabrous and lymph material is predominantly a human tissue and the viral molecules recovered are remnants of an active infection.

7. Line 104: Why is the small number of bacterial reads unexpected? Please explain Response: Ancient DNA libraries typically contain a large number of bacterial reads originating from soil or other environmental sources. In comparison to the majority of aDNA libraries, these vaccination kits contain a very small number of bacterial reads predominantly due to the absence of environmental contaminant bacterial sources. 8. Line 106: Please mention the enrichment method here.

Response: We have clarified that this is an enrichment for OPXV molecules and make reference to the Online Supplementary Materials. Given the length restrictions of the Short Report format, we cannot expand further within the main text.

9. 154: I would suggest to assemble also the enriched reads. For a draft analysis the quality should be sufficient and could provide further insights.

Response: We have previously attempted de novo assembly using enriched data and have found the outcome to be unpredictable. Given the biases associated with enrichment in general, and that our baits are designed across a wide variety of OPXV, not just VACV, we do not believe that this analysis will add to this work.

10. Figure 2a: The newly described CPXV from voles have to be also included (e.g. also the Ectromelia like CPXV from bank voles)

Response: While we agree that this is an interesting virus, as it is clearly a CPXV rather than a VACV strain, forming a distinct lineage, in reality it adds nothing to our understanding of the origin and evolution of the US Civil War vaccine strains. As we note in the paper, our phylogenetic analysis only includes representative strains.

11. Line 598: The metagenome analysis should be repeated and improved by using a modern metagenome workflow. This should be done to be sure that there is no further hits for viruses or bacteria.

Response: As poxviruses are the sole focus of this paper we are unsure how looking for additional pathogens would be helpful, and we are severely constrained by the length restrictions on Short Report papers. We can address this in a follow-up study.

**2^nd^ round**

**Reviewer #2**

I thank the author's for their response, but respectfully suggest that they have missed the point I was trying to make. It's my fault for not being clear and perhaps I was assuming everyone knows why a description of the telomere sequences is so critical for our understanding of this virus.

Nobody doubts the close sequence relationship between modern VAC strains, these old strains, and the Mongolian horsepox. All of the conserved cores of Orthopoxviruses look much alike and are easily aligned. However, what your readers would really like to know is how Jenner took a virus from the wild (commonly thought to be some larger VAC-like cowpox) and it then lost ~25kbp of non-essential sequence from the ends over the next century. As the authors note this produced what is now called vaccinia virus.

I am simply asking what unique sequences are found at the ends of the genome? Could they have all been retrieved? Are any not seen in modern vaccinia?

All of the telomeric sequences should have been captured as the enrichment strategy cast a pretty broad net. Unfortunately the authors have offered no discussion or description of genome elements that are far more critical for understanding vaccinia's origins than is the central core. If the assemblies are as contiguous as the authors claim they are, then they should encompass all of the ITR's. If fact the assemblies should be best there because these regions are diploid. Then they should be seeing contigs that either:

A) run from the first/last ORF in one of the modern vaccinia's into the 72/150bp repeats that mark the start of the non-coding ends of many Orthopoxviruses	OR

B) incorporate additional reads resembling sequences found in the telomeres of horsepox, cowpox or whatever other virus vaccinia derives from.

The 72 bp repeats comprise a barrier to further sequence assembly of course, but there's no reason why the rest of the genome should't join up to them with as much continuity as is seen elsewhere.

If we assume nothing got lost, then the presence of some additional sequences in the left and/or right ITRs would show that these strains had not yet fully evolved into a modern vaccinia. Their complete absence would show that (1) vaccinia arose even earlier in its passage history or (2) the model for gene loss is wrong. A virus with some partial retention of these sequences would be a "missing link" and a pointer to the ancestral strain(s). What the reader can't tell is whether the authors "got everything" that could be here, the quality of the connectivity in these (or any) regions, or what the sequence they did assemble looks like.

There's no need for more phylograms looking at the central core. That's not especially informative as it just reaffirms the well-known relationship between vaccinia and horsepox. If it is possible to answer my question it would make the paper far more interesting. It would address THE critically important question concerning the origins and evolutionary trajectory of Jenner's virus in the hands of humans.

Two other less critical issues:

- When discussing contig/genome sizes it is essential to explain whether one is talking about the assembled contig (usually one ITR plus the central core) or the presumed genome with the second ITR restored to the other end. That's not clear in the manuscript.

- A map showing the claimed contigs (e.g. 179,308 nt) plus the associated read depths would help the reader gain a much better understanding of just how good these assemblies are. The current written description is very vague and, of course, genome papers have a unique problem in that the reviewers get to see only the interpretation of data. Given the duplication of sequences in the ITRs one would like to see evidence that the read depth is also doubled in this region. This could be a supplemental file.

**Reviewer #3**

Most of the questions asked by the reviewers were solved or explained in more detail by the authors. Those clarifications improved the paper.

Especially the described de novo assembly of sequence information of one sample and the repeated GBlocks analysis with more relaxed settings are arguing against a severe filtering or alignment bias.

However, there are still some minor issues I would suggest to think about (respecting the space limitations):

- The discussion about horsepox virus and its relationship to vaccinia should still be more careful. The central genome part is very identical, but there are also clear differences looking at the whole genome (length, insertions/deletions, ancestral genome parts) which were described before. It should be therefore avoided to state that vaccinia is the same as horsepox virus. It would be good to make clear that the until today analysed horsepox viruses are closely related to but distinct from vaccinia (as shown in the cited references like Esparza et al.)

- There was no attempts to assemble the enriched reads. The reasons mentioned (bias due to the probes etc.) are difficult to follow since a very broad set of orthopoxvirus probes was used which should represent very well sequence variations. I would use the arguments against it after having it done and the results look not reliable. But not doing it is more like missing out on a chance.

- When no further metagenomics analysis is done, the limitations of the conclusions about the reported pathogen content should be stated in the manuscript.

**Response to reviewers**

**Reviewer #2**

I thank the author's for their response, but respectfully suggest that they have missed the point I was trying to make. It's my fault for not being clear and perhaps I was assuming everyone knows why a description of the telomere sequences is so critical for our understanding of this virus.

Nobody doubts the close sequence relationship between modern VAC strains, these old strains, and the Mongolian horsepox. All of the conserved cores of Orthopoxviruses look much alike and are easily aligned. However, what your readers would really like to know is how Jenner took a virus from the wild (commonly thought to be some larger VAC-like cowpox) and it then lost ~25kbp of non-essential sequence from the ends over the next century. As the authors note this produced what is now called vaccinia virus.

I am simply asking what unique sequences are found at the ends of the genome? Could they have all been retrieved? Are any not seen in modern vaccinia?

Response: We built our capture baits based on all available sequences (entire genomes) of existing VACV (and other Orthopoxvirus) sequences. If the DNA in our samples is similar we would have captured these sequences with our methodology. Completely novel sequences (at the termini or elsewhere) will not be captured and so would have to be recovered through shotgun sequencing alone. Despite our best attempts, de novo assemblies do not reconstruct any additional novel sequences at the termini of our ancient strains, and we are restricted by the limitations of the algorithm used in that reconstruction. Further attempts to map the data to various reference sequences including the cowpox and horsepox viruses show that there remain considerable gaps of coverage for our best sample VK01 relative to the respective reference even post-enrichment (Figures RR1-3). Furthermore, we disagree with the reviewers conjecture that VACV is derived by gene-loss from cowpox virus: there is no evidence for these statements. The virus that Jenner used is unknown, it might have been cowpox or vaccinia, or he may have used both viruses at different times. Without having the viruses, or their genomes, no one knows for sure. This statement is both fantasy and also not the point of this paper.

All of the telomeric sequences should have been captured as the enrichment strategy cast a pretty broad net. Unfortunately the authors have offered no discussion or description of genome elements that are far more critical for understanding vaccinia's origins than is the central core. If the assemblies are as contiguous as the authors claim they are, then they should encompass all of the ITR's. If fact the assemblies should be best there because these regions are diploid. Then they should be seeing contigs that either:

A) run from the first/last ORF in one of the modern vaccinia's into the 72/150bp repeats that mark the start of the non-coding ends of many Orthopoxviruses OR

B) incorporate additional reads resembling sequences found in the telomeres of horsepox, cowpox or whatever other virus vaccinia derives from. Response: As stated above, we can only report what the de novo assembly software produces. It does not reconstruct these telomeric regions as contiguous with our singular large 184,677bp contig. There are additional small contigs produced by the algorithm that are blast assigned to VACV. However, the SPAdes assembly is unable to place them within the larger contig and therefore the most prudent and conservative approach is to not create artificial genomic architecture since we remain uncertain as to their genomic placement or if they may represent short erroneously assembled genomic sequences. We also note that post-enrichment, the relative proportion of molecules recovered cannot be considered even across the genome (see Figures RR1-3). To include such data violates the assumptions of the de novo assembly algorithm. Hence, the reviewer is asking us for data that we cannot faithfully provide. The 72 bp repeats comprise a barrier to further sequence assembly of course, but there's no reason why the rest of the genome should't join up to them with as much continuity as is seen elsewhere.

If we assume nothing got lost, then the presence of some additional sequences in the left and/or right ITRs would show that these strains had not yet fully evolved into a modern vaccinia. Their complete absence would show that (1) vaccinia arose even earlier in its passage history or (2) the model for gene loss is wrong. A virus with some partial retention of these sequences would be a "missing link" and a pointer to the ancestral strain(s). What the reader can't tell is whether the authors "got everything" that could be here, the quality of the connectivity in these (or any) regions, or what the sequence they did assemble looks like.

Response: Again, we have produced the best ancient reconstructed genome given the limitations of ancient damaged DNA and the assembly software. We have not suggested that we “got everything”; in fact, we state clearly in our manuscript that we believe the de novo assembly of VK01 to be incomplete (lines 145-149) and that we there are genomic rearrangements present in some of the other samples (particularly VK02) that cannot be properly resolved through our consensus calling methods nor with de novo assembly. This however does NOT alter the results of our analysis in any way.

There's no need for more phylograms looking at the central core. That's not especially informative as it just reaffirms the well-known relationship between vaccinia and horsepox. If it is possible to answer my question it would make the paper far more interesting. It would address THE critically important question concerning the origins and evolutionary trajectory of Jenner's virus in the hands of humans.

Two other less critical issues:

- When discussing contig/genome sizes it is essential to explain whether one is talking about the assembled contig (usually one ITR plus the central core) or the presumed genome with the second ITR restored to the other end. That's not clear in the manuscript.

Response: We believe our assembly represents the central core and one, potentially partial, ITR, we have further emphasized this point in the manuscript at lines 149-151.

- A map showing the claimed contigs (e.g. 179,308 nt) plus the associated read depths would help the reader gain a much better understanding of just how good these assemblies are. The current written description is very vague and, of course, genome papers have a unique problem in that the reviewers get to see only the interpretation of data. Given the duplication of sequences in the ITRs one would like to see evidence that the read depth is also doubled in this region. This could be a supplemental file.

Response: We provide an example of such data in Figures RR1-3. As can be seen from the shotgun data, there is an elevated amount of coverage on the 3’ end of the VK01 de novo assembly. However, we note that this pattern of increased coverage in the same data mapped to the Copenhagen VACV, Horsepox, and GRI-90 cowpox reference sequences.

**Reviewer #3**

Most of the questions asked by the reviewers were solved or explained in more detail by the authors. Those clarifications improved the paper.

Especially the described de novo assembly of sequence information of one sample and the repeated GBlocks analysis with more relaxed settings are arguing against a severe filtering or alignment bias. However, there are still some minor issues I would suggest to think about (respecting the space limitations):

- The discussion about horsepox virus and its relationship to vaccinia should still be more careful. The central genome part is very identical, but there are also clear differences looking at the whole genome (length, insertions/deletions, ancestral genome parts) which were described before. It should be therefore avoided to state that vaccinia is the same as horsepox virus. It would be good to make clear that the until today analysed horsepox viruses are closely related to but distinct from vaccinia (as shown in the cited references like Esparza et al.)

Response: Yes, there are some genomic differences between horsepox and VACV and we don't say otherwise. The only point we really want to make is that these two viruses are closely related and we see no strong counter evidence against this statement.

- There was no attempts to assemble the enriched reads. The reasons mentioned (bias due to the probes etc.) are difficult to follow since a very broad set of orthopoxvirus probes was used which should represent very well sequence variations. I would use the arguments against it after having it done and the results look not reliable. But not doing it is more like missing out on a chance.

Response: We’re sorry we didn’t make this clearer. We did attempt de novo assemblies with the enriched reads. As we noted in our previous response, we found the behaviour of the assembly algorithm to be highly unpredictable when provided with enriched data. This is not surprising as enrichment, using a broad bait set composed of OPXV sequences, can skew the relative proportion of molecules sequenced meaning that, even given a target genome, all molecules are not necessarily sequenced with equal probability. This effect is evident in Figures RR1-3. In losing the equal probability of recovering ALL molecules, the de novo assembly algorithms lose important information enabling proper reconstruction as to the frequency in which a particular kmer is observed. This can lead to incorrect assemblies which are difficult to judge. As an example, while de novo assembly of VK01 using only shotgun data results in a VACV contig 184,677bp long, repeating the assembly using the shotgun data combined with the enriched data leads to a contig only 14,083bp long. The truncation is, presumably, due to the abundance of sequences present in varying proportions along the genome, causing the algorithm to be unable to determine how to correctly elongate the assembly. We repeated the de novo assemblies for the other samples which have a far lower proportion of shotgun data and are instead comprised on predominantly enriched data. This did lead to longer contigs for those samples relative to the assemblies produced from just shotgun data; again, however, we have no faith in the accuracy of those assemblies. We present a maximum likelihood tree produced from these assemblies in Figure RR4. The topology of this tree is the same as that presented in main text Figure 2, and in fact the final Gblocked alignment for the de novo assembled genomes is considerably shorter at only 95,420bp.

- When no further metagenomics analysis is done, the limitations of the conclusions about the reported pathogen content should be stated in the manuscript

Response: We have been careful throughout the manuscript to only discuss what can safely be concluded from the data. The metagenomic profiling of the libraries is discussed in lines 601-617.

**3^rd^ round**

**Reviewer #3**

The open questions of the reviewers were discussed by the authors and explained for a better understanding.
